# Molecular and cellular context influences *SCN8A* variant function

**DOI:** 10.1172/jci.insight.177530

**Published:** 2024-05-21

**Authors:** Carlos G. Vanoye, Tatiana V. Abramova, Jean-Marc DeKeyser, Nora F. Ghabra, Madeleine J. Oudin, Christopher B. Burge, Ingo Helbig, Christopher H. Thompson, Alfred L. George

**Affiliations:** 1 Department of Pharmacology, Northwestern University Feinberg School of Medicine, Chicago, Illinois, USA.; 2 Department of Biomedical Engineering, Tufts University, Medford, Massachusetts, USA.; 3 Department of Biology, Massachusetts Institute of Technology, Cambridge, Massachusetts, USA.; 4 Division of Neurology, Children’s Hospital of Philadelphia, Philadelphia, Pennsylvania, USA.; 5 Department of Neurology, Perelman School of Medicine, University of Pennsylvania, Philadelphia, Pennsylvania, USA.

**Keywords:** Genetics, Neuroscience, Epilepsy, Neurodevelopment, Sodium channels

## Abstract

Pathogenic variants in *SCN8A*, which encodes the voltage-gated sodium (Na_V_) channel Na_V_1.6, associate with neurodevelopmental disorders, including developmental and epileptic encephalopathy. Previous approaches to determine *SCN8A* variant function may be confounded by use of a neonatally expressed, alternatively spliced isoform of Na_V_1.6 (Na_V_1.6N) and engineered mutations rendering the channel tetrodotoxin (TTX) resistant. We investigated the impact of *SCN8A* alternative splicing on variant function by comparing the functional attributes of 15 variants expressed in 2 developmentally regulated splice isoforms (Na_V_1.6N, Na_V_1.6A). We employed automated patch clamp recording to enhance throughput, and developed a neuronal cell line (ND7/LoNav) with low levels of endogenous Na_V_ current to obviate the need for TTX-resistance mutations. Expression of Na_V_1.6N or Na_V_1.6A in ND7/LoNav cells generated Na_V_ currents with small, but significant, differences in voltage dependence of activation and inactivation. TTX-resistant versions of both isoforms exhibited significant functional differences compared with the corresponding WT channels. We demonstrated that many of the 15 disease-associated variants studied exhibited isoform-dependent functional effects, and that many of the studied *SCN8A* variants exhibited functional properties that were not easily classified as either gain- or loss-of-function. Our work illustrates the value of considering molecular and cellular context when investigating *SCN8A* variants.

## Abstract 2

The functional consequences of epilepsy-associated pathogenic variants in the voltage-gated sodium channel Nav1.6-encoding gene *SCN8A* are affected by splice isoform and molecular context.

## Graphical abstract

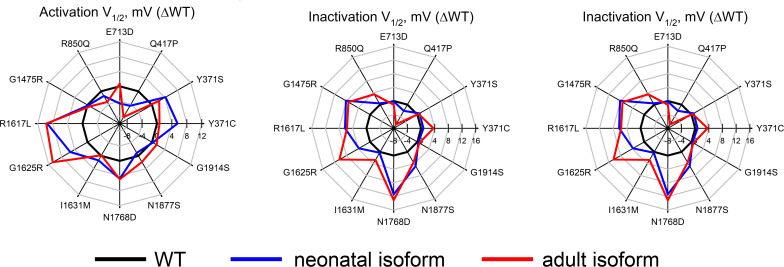

## Introduction

Pathogenic variants in genes encoding voltage-gated sodium (Na_V_) channels are frequently discovered in individuals with early-onset epilepsy, developmental and epileptic encephalopathy (DEE), and related neurodevelopmental disorders (NDDs) (1, 2). Determining the functional consequences of Na_V_ channel variants can provide information about pathophysiological mechanisms and potentially guide precise therapeutic approaches (3, 4). Using the correct molecular context (e.g., species origin, splice isoform) for investigating function of an ion channel variant is vital for an accurate assessment.

Pathogenic variants in *SCN8A*, which encodes Na_V_1.6, have emerged as important causes of neurodevelopmental disorders, with typical onset during infancy (5). The earliest discoveries associated DEE with nontruncating variants having gain-of-function properties (e.g., enhanced persistent current, altered voltage dependence of activation). Subsequently, *SCN8A* variants were discovered in individuals affected with epilepsy having a wider spectrum of clinical severity as well as NDD without seizures (6).

In mature neurons, Na_V_1.6 is localized to the axon initial segment where the channel serves to initiate action potentials (7). The gene undergoes specific alternative splicing events during early development, including the in-frame inclusion of 1 of 2 distinct versions of exon 5 that encodes a portion of the first voltage-sensing domain (8). Exon 5N dominates during embryonic development and immediately after birth, but around 1 year of age transcripts containing the alternative exon 5A surpass those containing 5N, and the 5A isoform becomes predominant in later childhood through adulthood (9). Importantly, the *SCN8A* reference coding sequence designated as variant 1 (NM_014191) by the National Center for Biotechnology Information (NCBI) includes exon 5N, whereas the sequence including exon 5A is curated as variant 3 (NM_001330260). These annotations guided the early annotation of *SCN8A* variants by genetic testing laboratories such that pathogenic variants in exon 5A were initially overlooked (10).

Previous studies of *SCN8A* variant function used a variety of expression systems, including rodent Na_V_1.6 (11–13) or the human channel with exon 5N (neonatal splice isoform) (14–19), which does not represent the most abundant splice isoform present during most of childhood and older (9). In some of these studies, Na_V_1.6 was expressed in a neuronal cell line (ND7/23) that exhibits large endogenous sodium currents. To discriminate between the endogenous and heterologously expressed Na_V_ channels in these cells, many investigators exploited pore domain mutations of tyrosine-371 (Y371C or Y371S) to render Na_V_1.6 insensitive to tetrodotoxin (TTX) (11–13, 16–21). However, this strategy could conceivably alter the functional impact of disease-associated variants, especially those located near the TTX interaction site in the channel structure. The heterogeneity in experimental approaches and recombinant Na_V_1.6 channel constructs limits the comparisons of variant dysfunction across laboratories. A uniform experimental approach free from the potential confounds of endogenous currents and second-site mutations that also considers the most relevant splice isoform would be valuable.

In this study, we analyzed a series of *SCN8A* variants using automated patch clamp recording of recombinant human Na_V_1.6 heterologously expressed in a derivative of ND7/23 neuronal cells that have low levels of endogenous Na_V_ current. We compared the functional properties of variants in the 2 isoforms generated by alternative splicing of exon 5 and used the relevant splice isoform to study a subset of variants discovered within exon 5A. Finally, we illustrate the importance of controlling for splice isoform by the functional study of an epilepsy-associated *SCN8A* haplotype with 2 de novo missense variants of uncertain significance within or outside exon 5N. Our findings demonstrate the importance of considering molecular and cellular context when investigating the functional consequences of *SCN8A* variants.

## Results

We initially expressed recombinant WT human Na_V_1.6 in HEK293T cells to evaluate efficiency and fidelity of automated patch clamp as a platform for evaluating the functional consequences of *SCN8A* variants. Results from these initial trials proved unsatisfactory because of inconsistent cell capture and suboptimal membrane seal formation. Therefore, we explored alternative cell lines, including the rat dorsal root ganglion neuron/mouse neuroblastoma hybrid ND7/23 cell line (22), which was used successfully by other groups for studying Na_V_1.6 variants (11–13, 16–19, 21). Because ND7/23 cells exhibit a large endogenous fast-gating Na_V_ current, previous studies utilized a TTX-resistant mutant form of Na_V_1.6 coupled with recording in the presence of TTX to isolate activity of the transfected channel. We chose an alternative approach designed to eliminate endogenous Na_V_ currents in ND7/23 cells, which obviates the need for TTX and non-native Na_V_1.6 sequences that could confound analysis of disease-associated mutations.

### Generation of a neuronal cell line with minimal Na_V_ current.

**Figure 1:**
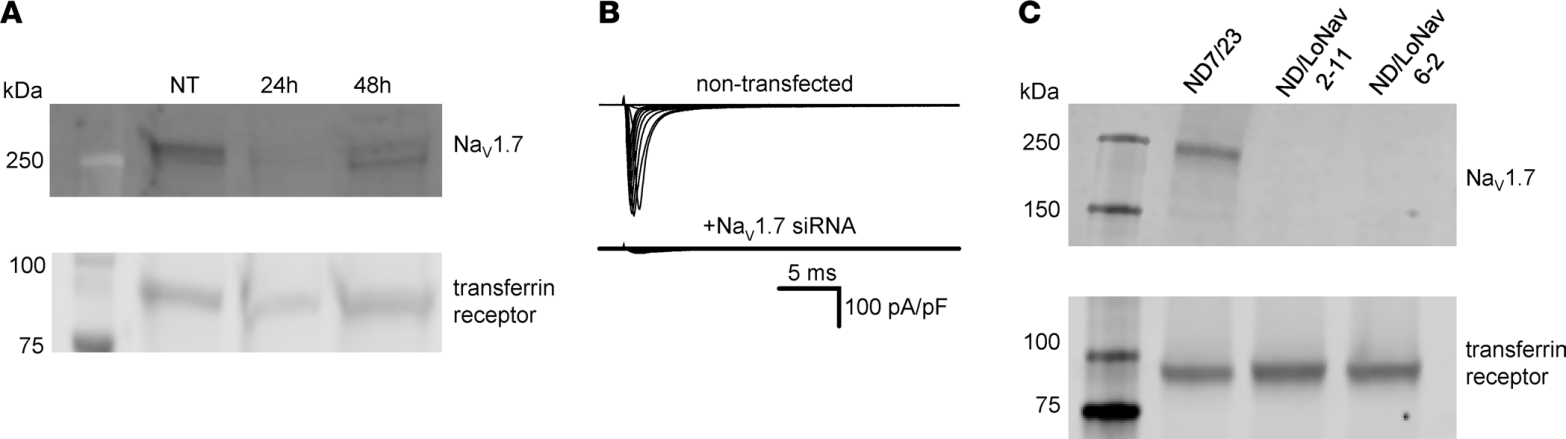
Na_V_1.7 siRNA reduces whole-cell current and Na_V_1.7 protein expression in ND7/23 cells. (**A**) Immunoblots of Na_V_1.7 and transferrin receptor (loading control) isolated from nontransfected (NT) and Na_V_1.7 siRNA–transfected (24 and 48 hours after transfection) ND7/23 cells. (**B**) Average whole-cell currents from nontransfected (*n* = 3) and Na_V_1.7 siRNA–transfected (*n* = 4) ND7/23 cells recorded by manual patch clamp. (**C**) Immunoblots of Na_V_1.7 and transferrin receptor isolated from ND7/23 and 2 clonal ND7/LoNav cell lines.

Previously reported transcriptome analyses (23, 24) and pharmacological studies (25) deduced that Na_V_1.7 was responsible for most of the endogenous Na_V_ current in ND7/23 cells. As additional proof, we transfected ND7/23 cells with siRNA targeting conserved sequences shared by rat and mouse Na_V_1.7, and then assessed knockdown success by immunoblotting and manual patch clamp recording. Transient knockdown of Na_V_1.7 eliminated immunodetectable protein and lowered endogenous Na_V_ current substantially (Figure 1, A and B). In separate experiments, we did not detect Na_V_1.6 protein in ND7/23 cell lysates using 2 different polyclonal antibodies validated against mouse brain (Supplemental Figure 1; supplemental material available online with this article; https://doi.org/10.1172/jci.insight.177530DS1), indicating that the low level of *Scn8a* mRNA detected in ND7/23 cells (23, 24) likely does not contribute to functional Na_V_ channel expression.

**Figure 2:**
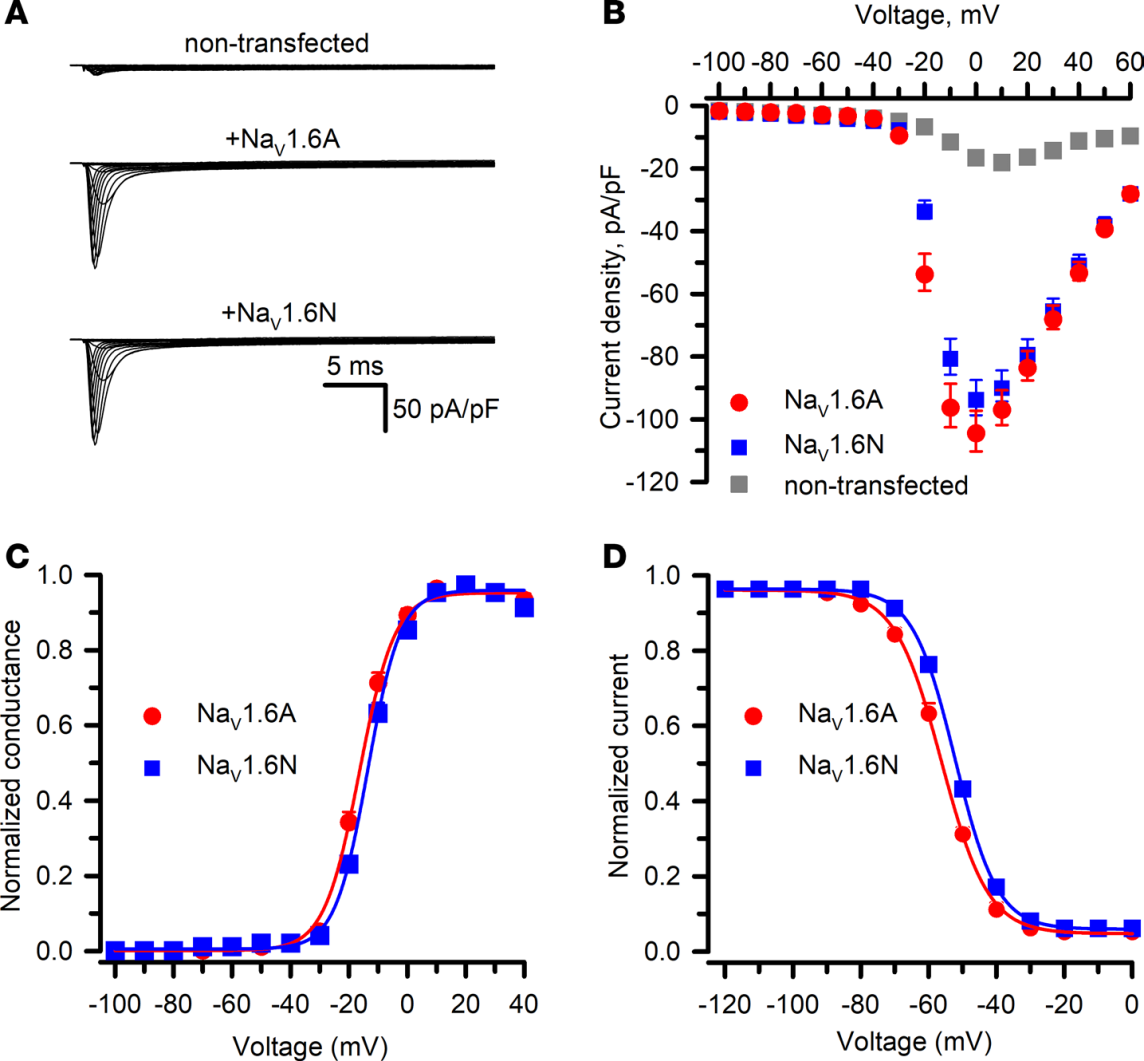
Functional properties of Na_V_1.6N and Na_V_1.6A expressed in ND7/LoNav cells. (**A**) Average whole-cell currents recorded using automated patch clamp from nontransfected ND7/LoNav cells or transiently transfected with Na_V_1.6A or Na_V_1.6N. In these experiments, transfection efficiency of WT Na_V_1.6A and Na_V_1.6N averaged 78.3% ± 3.5% and 74.4% ± 5.1%, respectively, and approximately 65% of wells in each 384-well plate exhibited cell capture and formation of stable high resistance (≥0.5 GΩ) membrane seals. (**B**) Average current density versus voltage measured from nontransfected ND7/LoNav cells (gray squares, *n* = 103), Na_V_1.6A (red circles, *n* = 90), or Na_V_1.6N (blue squares, *n* = 86). (**C**) Conductance-voltage plots recorded from ND7/LoNav cells expressing Na_V_1.6A (red circles, *n* = 96) or Na_V_1.6N (blue squares, *n* = 90). (**D**) Voltage dependence of inactivation recorded from ND7/LoNav cells expressing Na_V_1.6A (red circles, *n* = 139) or Na_V_1.6N (blue squares, *n* = 142). Quantitative data with statistical comparisons are provided in Supplemental Data Set 1.

For stable suppression of endogenous Na_V_ current in ND7/23 cells, we employed CRISPR/Cas9 genome editing to disrupt the coding sequences of rat and mouse Na_V_1.7 (see Methods). An initial round of gene editing yielded clonal lines with multiple frame-shifting deletions in both rat and mouse Na_V_1.7, but a small subpopulation of channel sequences with in-frame deletions was also observed. A second round of gene editing was successful in introducing frame shifts in all detectable Na_V_1.7 transcripts. Two clones with negligible endogenous Na_V_1.7 protein levels (Figure 1C) and small endogenous inward currents (peak current density less than 15 pA/pF; Figure 2, A and B) were selected. The final clonal cell line (designated ND7/LoNav) was efficiently electroporated with human Na_V_1.6 and proved amenable to high-throughput automated patch clamp analysis. We did not observe overt differences in cell morphology between original and derivative cell lines.

### Na_V_1.6 splice isoforms exhibit distinct functional properties.

We compared the functional properties of adult (Na_V_1.6A) and neonatal (Na_V_1.6N) splice isoforms expressed in ND7/LoNav cells. Cells expressing either Na_V_1.6A or Na_V_1.6N exhibited large, voltage-activated inward currents with similar peak current densities, which were approximately 6-fold larger and had distinct biophysical properties compared with currents recorded from nontransfected cells (Figure 2, A and B, and Supplemental Data Set 1). Cells expressing either Na_V_1.6 isoform generated whole-cell currents with similar inactivation kinetics; however, there were small but significant differences between Na_V_1.6A and Na_V_1.6N in the voltage dependence of activation (Supplemental Data Set 1). Specifically, Na_V_1.6A exhibited a hyperpolarized V_1/2_ (difference of –2.9 ± 0.5 mV) and steeper voltage dependence of activation (Figure 2C) along with a hyperpolarized voltage dependence of inactivation (V_1/2_ difference of –3.4 ± 0.4 mV) (Figure 2D) relative to Na_V_1.6N. These results indicate that Na_V_1.6A and Na_V_1.6N exhibit distinct biophysical properties, and suggest that the functional consequences of disease-associated Na_V_1.6 variants may be influenced by the splice isoform.

### Functional consequences of disease-associated SCN8A variants.

We determined the functional properties of 8 disease-associated *SCN8A* missense variants (Q417P, R850Q, G1475R, R1617L, G1625R, I1631M, N1768D, and N1877S) and 2 ultrarare variants of uncertain significance reported in the ClinVar database (https://www.ncbi.nlm.nih.gov/clinvar/) (Q713D and G1914S) in both Na_V_1.6A and Na_V_1.6N. Five variants (R850Q, G1475R, R1617L, G1625R, and N1877S) are recurrent, and 3 (R850Q, G1475R, and R1617L) were previously investigated to determine mutation-associated functional effects (13, 15–17, 26). Three nonrecurrent variants were Q417P, which was found in a female with intractable infantile spasms and global developmental delay (also reported in ref. 27), I1631M discovered in a male with generalized epilepsy with onset at age 5 months followed in later childhood by partial epilepsy and mild intellectual disability, and N1768D (11). We investigated the functional consequences of 3 other disease-associated variants discovered in exon 5A (V211R, R223G, and I231T) (10) only in Na_V_1.6A. A summary of clinical phenotypes associated with each variant is presented in Supplemental Table 1. We also investigated the functional properties of 2 engineered TTX-resistance mutations (Y371C and Y371S) in both Na_V_1.6A and Na_V_1.6N. The location of all studied variants on the Na_V_1.6 protein are illustrated in Supplemental Figure 2. For this study, we analyzed electrophysiological data from 3,884 cells.

Variant-specific effects on channel properties were determined by quantifying differences relative to isoform-matched WT channels that were expressed and recorded in parallel. We analyzed current density, time constant (τ) of fast inactivation at 0 mV, voltage dependence of activation and inactivation, window current, frequency-dependent loss of channel availability (i.e., current rundown measured at 20 Hz), recovery from fast inactivation, persistent current, and net charge movement during slow voltage ramps. Averaged whole-cell currents normalized to peak isoform-matched WT current are presented in Supplemental Figures 3 and 4 for variants expressed in Na_V_1.6N and Na_V_1.6A, respectively.

**Figure 3:**
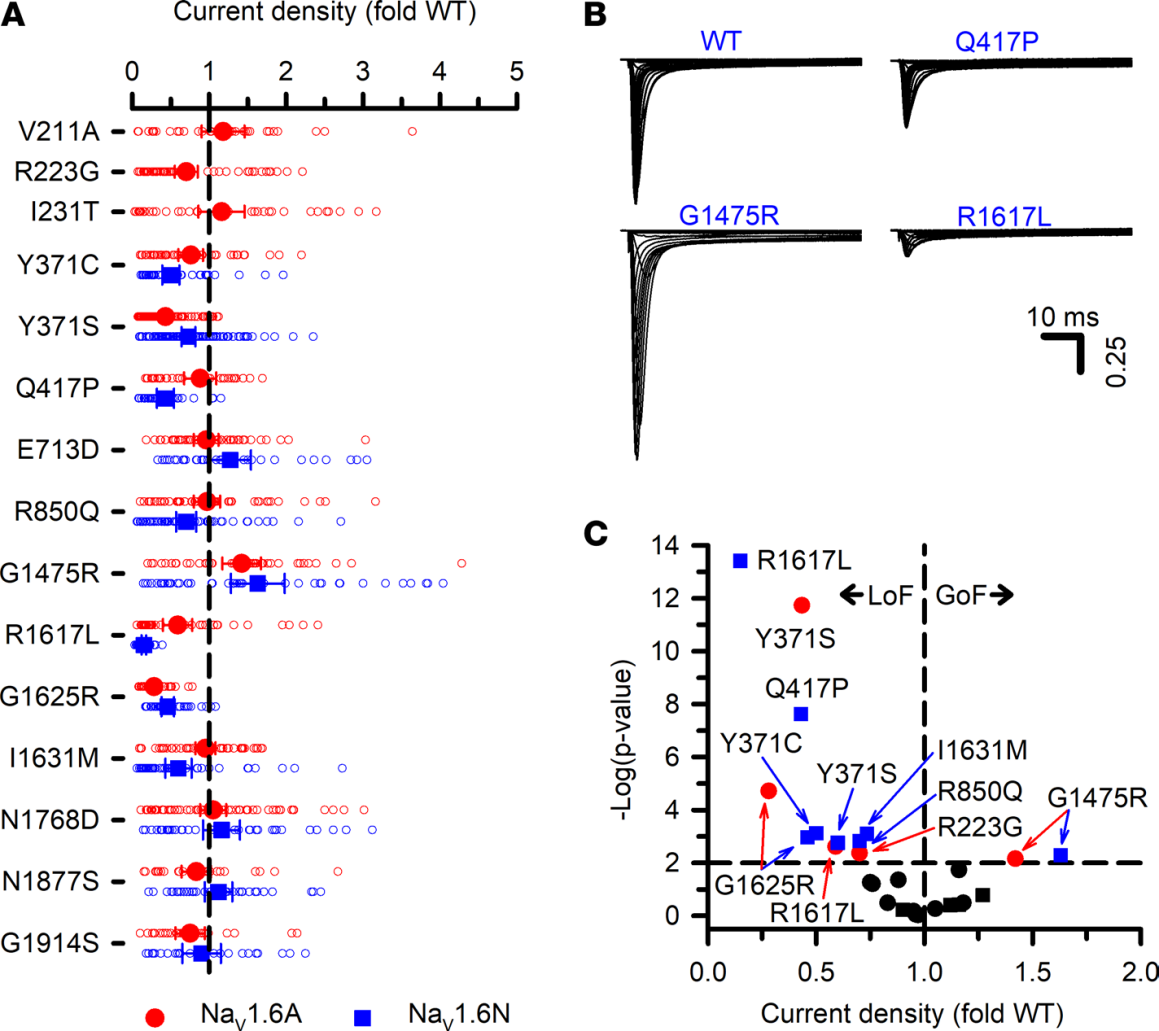
Current density of Na_V_1.6 variants. (**A**) Peak whole-cell current density for Na_V_1.6 variants displayed as fold difference from WT channels recorded in parallel. All individual data points are plotted as open symbols and mean values are shown as larger filled symbols (*n* = 35–108 per variant). Error bars represent 95% CI. Data from Na_V_1.6A or Na_V_1.6N are indicated as red or blue symbols, respectively. Values to the right or left of the vertical dashed line (normalized WT value) represent current density larger or smaller than WT, respectively. (**B**) Averaged current traces, normalized to WT peak current density, for WT Na_V_1.6N and select variants with either larger (G1475R) or smaller (Q417P) current density. (**C**) Volcano plot of mean values highlighting variants with peak current density significantly (*P* < 0.01, horizontal dashed line) different from WT. Symbols to the left of the vertical dashed line denote smaller current (loss of function, LoF), while symbols to the right indicate larger current (gain of function, GoF). Black symbols represent variants with no significant difference from WT. Quantitative data with statistical comparisons are provided in Supplemental Data Set 2 (Na_V_1.6N) and Supplemental Data Set 3 (Na_V_1.6A).

**Figure 4:**
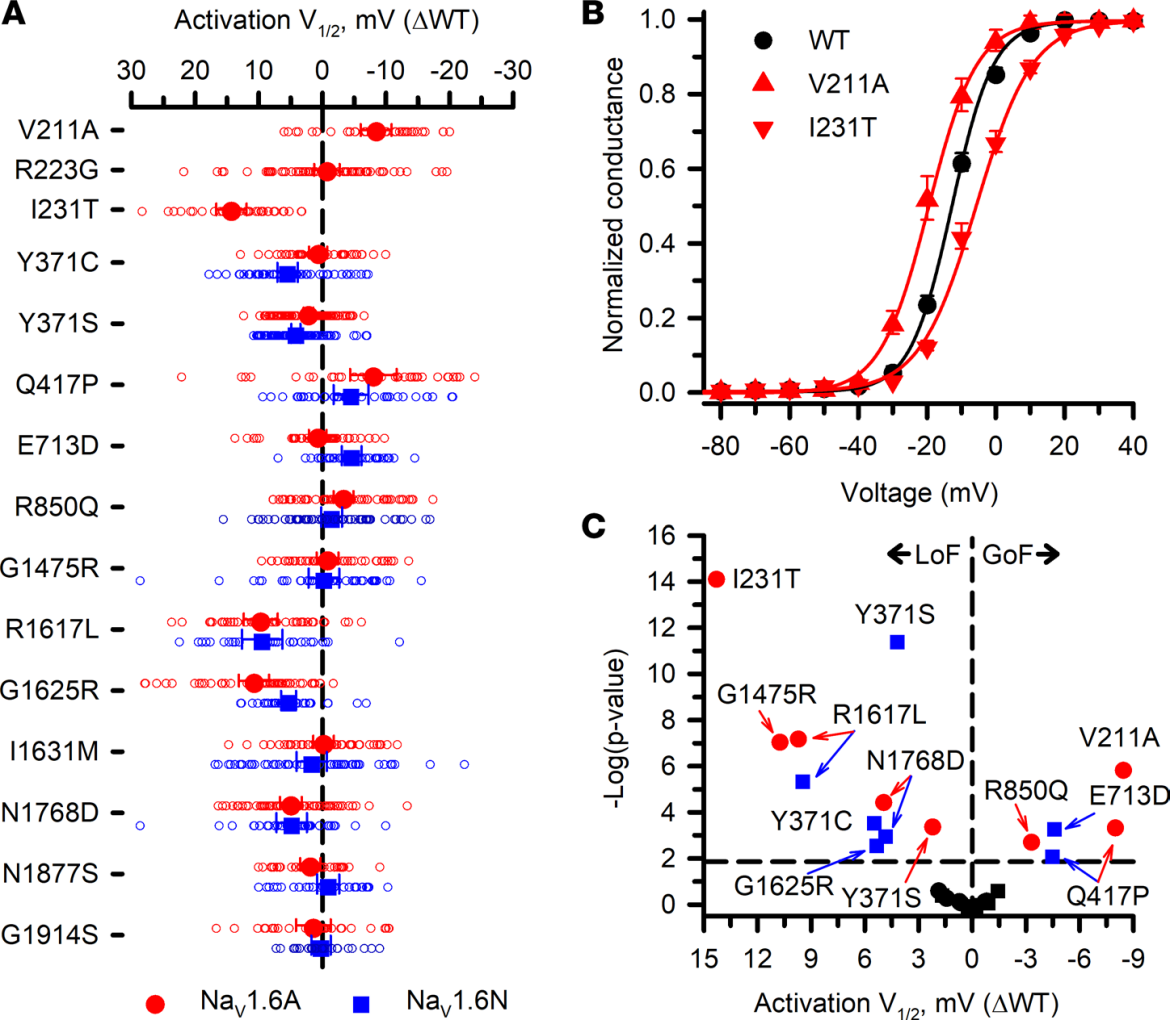
Voltage dependence of activation for Na_V_1.6 variants. (**A**) Averaged voltage dependence of activation V_1/2_ obtained from fitting the data for each variant-expressing cell and plotted as difference (ΔV_1/2_ in mV) from the averaged V_1/2_ for WT channels recorded in parallel. All individual data points are plotted as open symbols and mean values are shown as larger filled symbols (*n* = 22–77 per variant). Error bars represent 95% CI. Data from Na_V_1.6A or Na_V_1.6N are indicated as red or blue symbols, respectively. Values to the right or left of the vertical dashed line (no difference from WT) indicate hyperpolarized (gain of function) or depolarized (loss of function) activation V_1/2_, respectively. (**B**) Conductance-voltage relationships for select variants expressed in Na_V_1.6A (red lines) illustrating hyperpolarized (V211A) or depolarized (I231T) shifts in activation V_1/2_ relative to WT channels (black line) recorded in parallel. (**C**) Volcano plot of mean values highlighting variants with significantly different (*P* < 0.01, horizontal dashed line) activation V_1/2_. Symbols to the left of the vertical dashed line denote depolarized V_1/2_ values (loss of function, LoF), while symbols to the right indicate hyperpolarized V_1/2_ values (gain of function, GoF). Black symbols represent variants with no significant difference from WT. Quantitative data with statistical comparisons are provided in Supplemental Data Set 2 (Na_V_1.6N) and Supplemental Data Set 3 (Na_V_1.6A).

**Figure 5:**
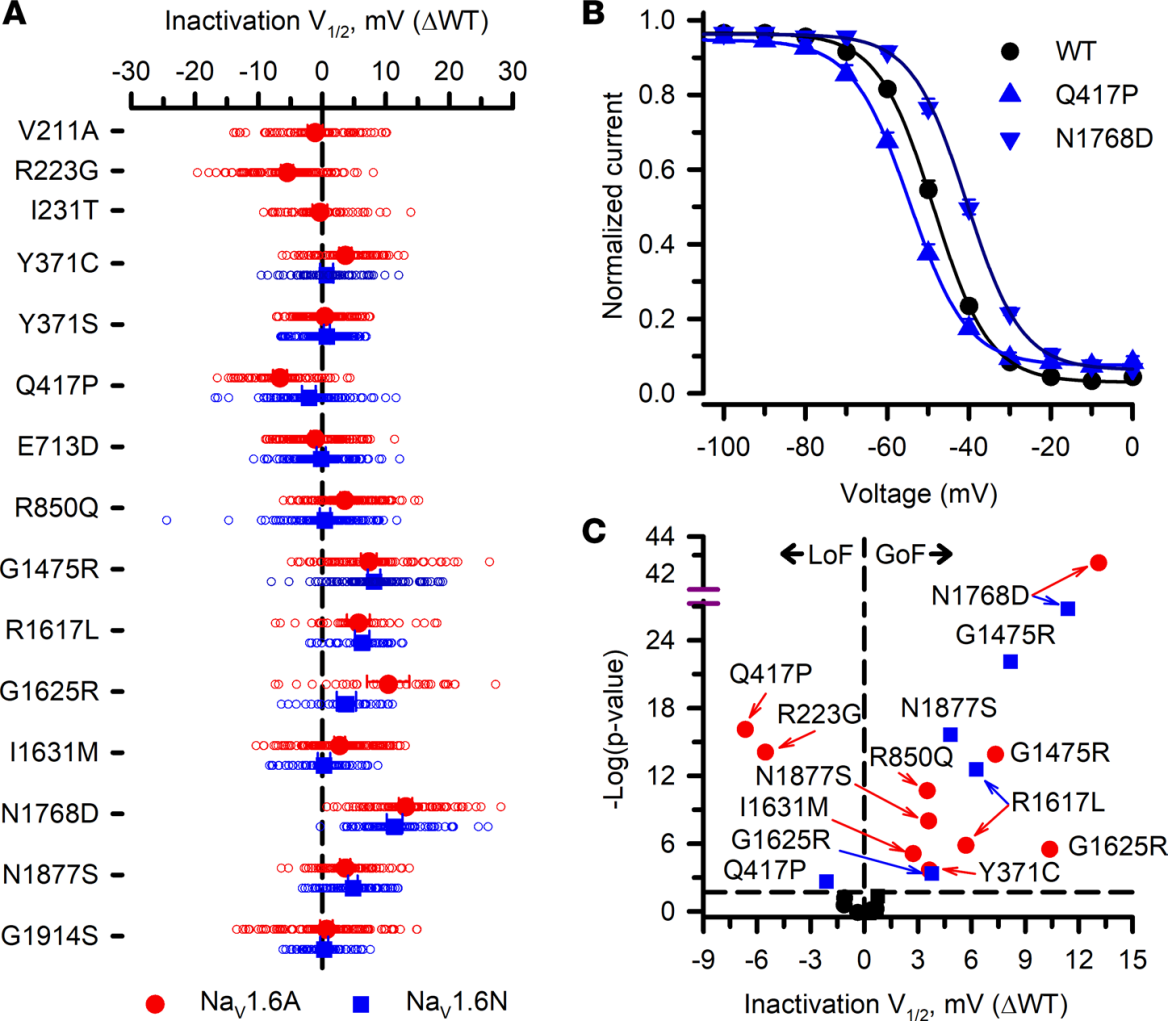
Voltage dependence of inactivation for Na_V_1.6 variants. (**A**) Averaged voltage dependence of inactivation V_1/2_ obtained from fitting the data for each variant-expressing cell and plotted as difference (ΔV_1/2_ in mV) from the averaged V_1/2_ for WT channels recorded in parallel. All individual data points are plotted as open symbols and mean values are shown as larger filled symbols (*n* = 29–142 per variant). Error bars represent 95% CI. Data from Na_V_1.6A or Na_V_1.6N are indicated as red or blue symbols, respectively. Values to the right or left of the vertical dashed line (no difference from WT) indicate depolarized (gain of function) or hyperpolarized (loss of function) inactivation V_1/2_, respectively. (**B**) Steady-state inactivation curves for select variants expressed in Na_V_1.6N (blue lines) illustrating hyperpolarized (Q417P) or depolarized (N1768D) inactivation V_1/2_ relative to WT channels (black line) recorded in parallel. (**C**) Volcano plot of mean values highlighting variants with significantly different (*P* < 0.01, horizontal dashed line) inactivation V_1/2_. Symbols to the left of the vertical dashed line denote hyperpolarized V_1/2_ values (loss of function, LoF), while symbols to the right indicate depolarized V_1/2_ values (gain of function, GoF). Black symbols represent variants with no significant difference from WT. Quantitative data with statistical comparisons are provided in Supplemental Data Set 2 (Na_V_1.6N) and Supplemental Data Set 3 (Na_V_1.6A).

Cells expressing most variants exhibited robust current, with the exception of R1617L expressed in the Na_V_1.6N splice isoform (Supplemental Data Sets 2 and 3). Measurable current density was significantly smaller than isoform-matched WT for 3 variants in the adult isoform and 7 in the neonatal isoform. Only 1 variant (G1475R) exhibited larger current density (Figure 3), and the increase was similar in both isoforms. Activation-voltage dependence varied considerably among this cohort of variants, with some exhibiting significantly depolarized or significantly hyperpolarized conductance-voltage relationships (Figure 4). Similarly, the voltage dependence of inactivation was significantly depolarized, significantly hyperpolarized, or WT-like (Figure 5). The overlap between the activation and inactivation curves illustrated in Figure 4B and Figure 5B, respectively, can be quantified as window current (Supplemental Figure 5), which defines a voltage range in which channels are activated but not inactivated. Most variants exhibited a significantly larger window current consistent with gain of function, whereas only 1 variant (I231T) had a significantly smaller window current.

**Figure 6:**
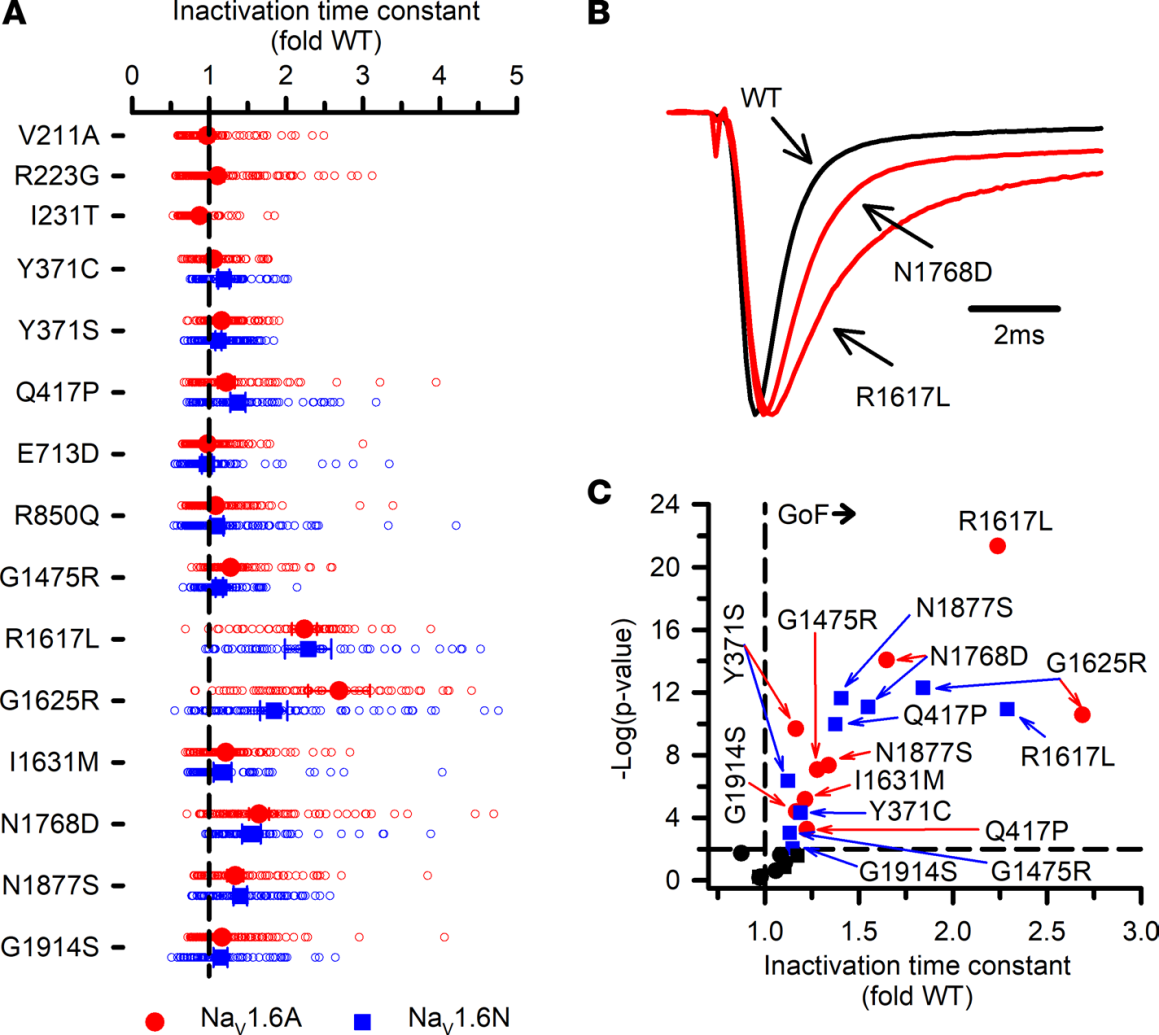
Inactivation kinetics for Na_V_1.6 variants. (**A**) Averaged inactivation time constants measured at 0 mV obtained by fitting current decay for each variant cell with a single exponential function and expressed as a ratio to the averaged WT channel value recorded in parallel. All individual data points are plotted as open symbols and mean values are shown as larger filled symbols (*n* = 57–160 per variant). Error bars represent 95% CI. Data from Na_V_1.6A or Na_V_1.6N are indicated as red or blue symbols, respectively. Values to the right or left of the vertical dashed line (average normalized WT value) indicate slower (gain of function) or faster (loss of function) inactivation kinetics, respectively. (**B**) Averaged traces recorded at 0 mV, normalized to the peak current density for select variants illustrating faster (V211A) or slower (N1768D) inactivation. (**C**) Volcano plot of mean values highlighting variants with significantly different (*P* < 0.01, horizontal dashed line) inactivation time constants. Symbols to the right of the vertical dashed line represent slower inactivation kinetics (gain of function, GoF). No variants exhibited significantly faster inactivation. Black symbols represent variants with no significant difference from WT. Quantitative data with statistical comparisons are provided in Supplemental Data Set 2 (Na_V_1.6N) and Supplemental Data Set 3 (Na_V_1.6A).

**Figure 7:**
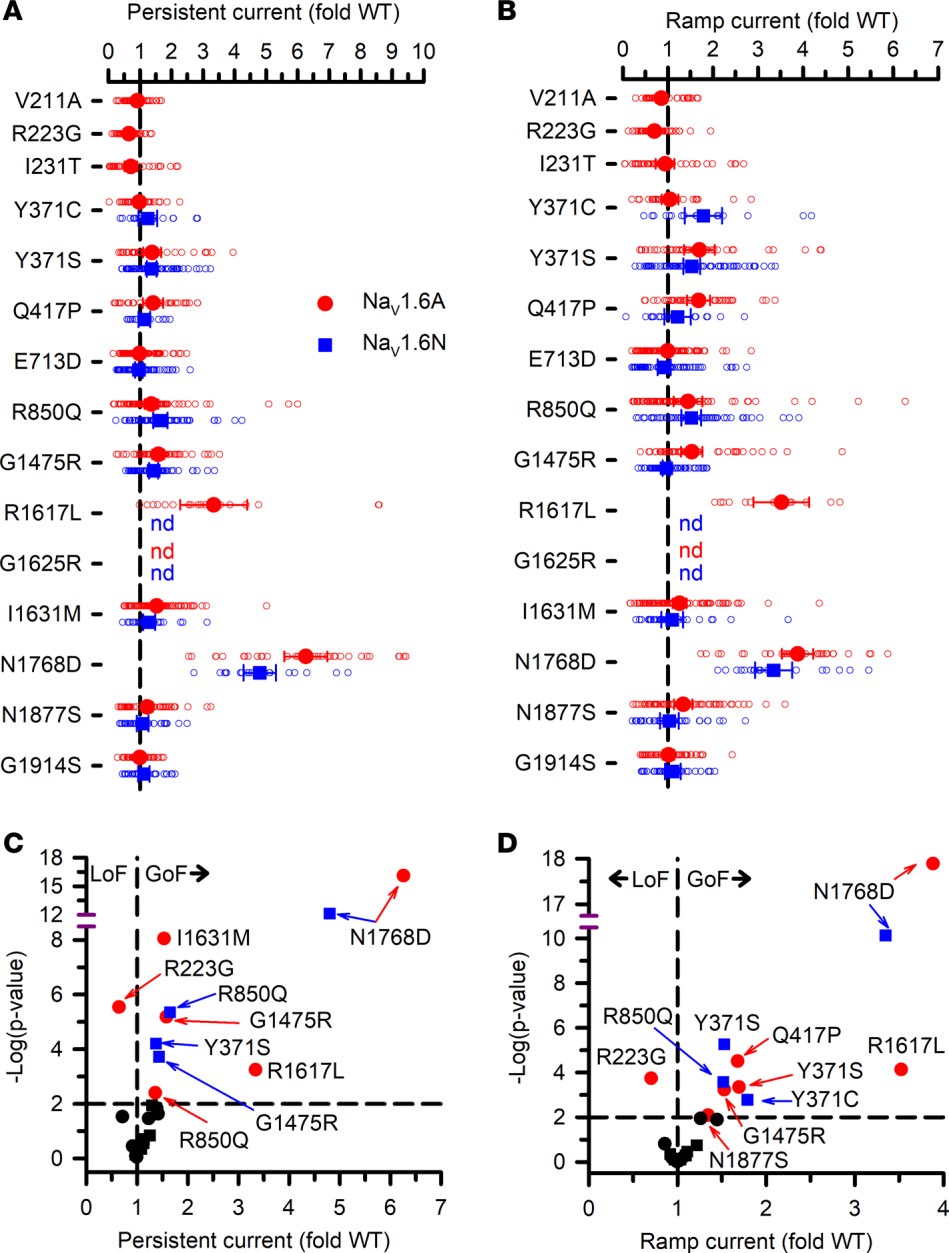
Persistent and ramp currents for Na_V_1.6. (**A** and **B**) Average persistent current amplitude (**A**) or net charge movement during a depolarizing voltage ramp (**B**) for variants displayed as fold differences from WT channels recorded in parallel. All individual data points are plotted as open symbols and mean values are shown as larger filled symbols (*n* = 9–94 per variant). Values for some variants were not determined (nd) because whole-cell current density was too small. Error bars represent 95% CI. Data from Na_V_1.6A or Na_V_1.6N are indicated as red or blue symbols, respectively. Values to the right or left of the vertical dashed lines (average normalized WT values) indicate larger (gain of function) or smaller (loss of function) currents, respectively. (**C** and **D**) Volcano plots of mean values highlighting variants with significantly different (*P* < 0.01, horizontal dotted line) levels of persistent current (**C**) or ramp current (**D**) compared with WT channels. Symbols to the left of the vertical dashed lines denote smaller current levels (loss of function, LoF), while symbols to the right indicate larger current levels (gain of function, GoF). Black symbols represent variants with no significant difference from WT. Quantitative data with statistical comparisons are provided in Supplemental Data Set 2 (Na_V_1.6N) and Supplemental Data Set 3 (Na_V_1.6A).

Inactivation kinetics (measured at 0 mV), persistent current, and ramp current amplitudes were also variable, with notable slowing of inactivation time course and greater persistent or ramp current for multiple variants (Figure 6 and Figure 7), which are consistent with gain of function. Only R223G exhibited smaller persistent or ramp current than WT. Persistent current was most prominent for the epilepsy-associated variant N1768D, in agreement with previous reports (11, 14). The kinetics observed for recovery from inactivation (Supplemental Figure 6) and frequency-dependent current rundown (Supplemental Figure 7) illustrate differences for some variants that are consistent with loss of function. The variants with the slowest recovery from inactivation were N1768D and Q417P expressed in either Na_V_1.6N or Na_V_1.6A, and this correlated with variable degrees (5%–13%) of frequency-dependent current rundown measured at 20 Hz.

Our investigation of the functional consequences of *SCN8A* using the 2 alternatively spliced versions of the channel revealed several variants with significant isoform-dependent properties. For a given variant, the magnitude of difference compared with the isoform-matched WT channel can be greater in either Na_V_1.6N or Na_V_1.6A (summarized in radar plots, Supplemental Figure 8). For example, the recurrent variant G1625R exhibits a greater degree of inactivation slowing and larger shifts in activation and inactivation V_1/2_ in Na_V_1.6A, whereas the degree of dysfunction was less severe in the neonatal isoform.

Our results demonstrated that disease-associated Na_V_1.6 variants exhibited various biophysical defects consistent with either gain of function (e.g., slower and less complete inactivation, hyperpolarized voltage dependence of inactivation) or loss of function (depolarized voltage dependence of activation, slower recovery from inactivation). However, opposing functional properties were observed for specific variants and this confounded the assignment of an overall effect using a simple binary classification scheme. We summarized the functional properties assessed for each variant expressed in either Na_V_1.6N or Na_V_1.6A in Supplemental Table 2, and generated heatmaps that scaled each measured parameter along a functional axis from loss to gain (Supplemental Figure 9). The heatmaps illustrate that most of the Na_V_1.6 variants exhibit complex patterns of dysfunction, a pattern also observed for *SCN2A* (Na_V_1.2) variants (28). The 2 *SCN8A* variants of uncertain significance (Q713D and G1914S) we studied exhibited minimal to no functional perturbations.

### Functional effects of a TTX-resistant variant.

An advantage of using ND7/LoNav cells is the ability to express a Na_V_ channel in a neuronal cell environment without requiring a pharmacological strategy to negate endogenous current. Because engineering TTX resistance has been widely used to distinguish Na_V_1.6 current from endogenous Na_V_ current in the original ND7/23 cell line, we examined functional properties of 2 TTX-resistant variants, Y371C and Y371S (11–13, 16–21) expressed in ND7/LoNav cells. Expression of Y371C or Y371S gave rise to measurable voltage-dependent Na_V_ currents in both Na_V_1.6N and Na_V_1.6A, but there were significant and heterogeneous differences in functional properties compared with cells expressing the isoform-matched WT channel. Current density was significantly smaller and the voltage dependence of activation was significantly depolarized for Y371S in both isoforms and for Y371C in Na_V_1.6A only (Figures 3 and 4). Recovery from inactivation was significantly slower for both Na_V_1.6N-Y371C and Na_V_1.6N-Y371S, but was WT-like for Na_V_1.6A-Y371S and significantly faster for Na_V_1.6A-Y371C (Supplemental Figure 6). The voltage dependence of inactivation was significantly depolarized only for Na_V_1.6A-Y371C (Figure 5). We also observed significant differences in inactivation kinetics between isoform-matched WT channels and cells expressing either TTX-resistant variant (Figure 6). Our findings indicated that TTX-resistant variants of Y371 affect the function of Na_V_1.6 in our experimental system.

### Value of studying variants in Na_V_1.6 splice isoforms.

**Figure 8:**
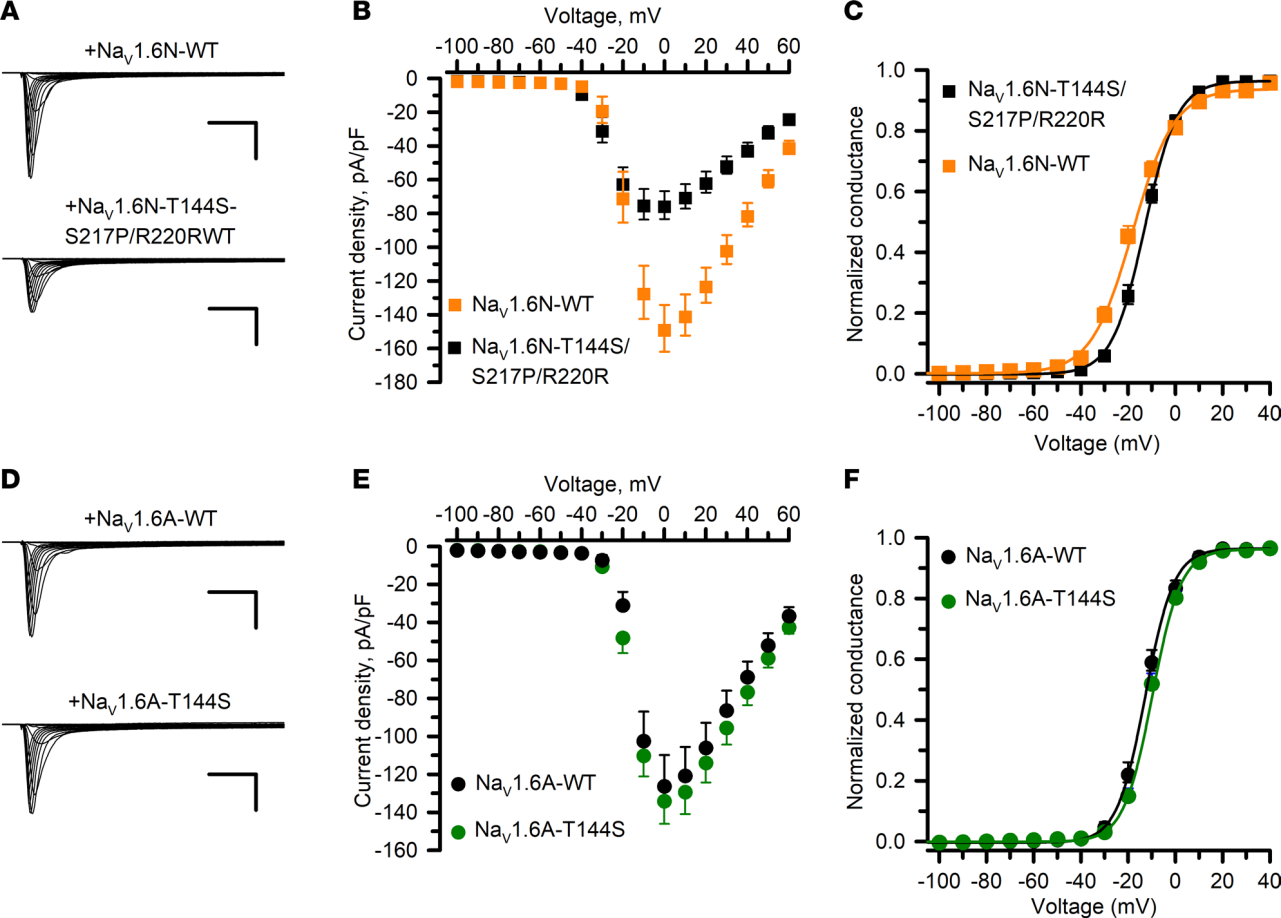
Functional properties of a complex *SCN8A* genotype of uncertain significance. (**A**) Averaged whole-cell current density recorded from ND7/LoNav cells expressing WT or variant Na_V_1.6N channels (*n* = 71–78 per variant). Scale bars: 5 ms (horizontal) and 25 pA/pF (vertical). (**B**) Current-voltage plots comparing WT (black symbols and lines) and variant (orange symbols and lines) Na_V_1.6N channels. Current amplitudes were normalized to cell capacitance to determine current density. (**C**) Conductance-voltage plots for WT (black symbols and lines) and variant (orange symbols and lines) Na_V_1.6N channels. (**D**) Averaged whole-cell current density recorded from ND7/LoNav cells expressing WT or variant Na_V_1.6A channels (*n* = 51–75 per variant). Scale bars: 5 ms (horizontal) and 50 pA/pF (vertical). (**E**) Current-voltage plots comparing WT (black symbols and lines) and variant (green symbols and lines) Na_V_1.6A channels. Current amplitudes were normalized to cell capacitance to determine current density. (**F**) Conductance-voltage plots for WT (black symbols and lines) and variant (green symbols and lines) Na_V_1.6A channels. Quantitative data with statistical comparisons are provided in Supplemental Data Set 2 (Na_V_1.6N) and Supplemental Data Set 3 (Na_V_1.6A). Error bars in panels **B**, **C**, **E**, and **F** represent 95% CI.

To further emphasize the importance of studying disease-associated variants in the most relevant Na_V_1.6 splice isoform, we demonstrated the functional consequences of a complex *SCN8A* genotype discovered in a female infant with early-onset DEE. The affected individual exhibited focal seizures and infantile spasms beginning at age 3 months, which were largely refractory to multiple drug treatments. She later developed tonic seizures, hypotonia, and cortical visual impairment. Clinical genetic testing identified 2 de novo missense variants (c.431C>G, p.T144S; c.649T>C, p.S217P). Subsequent long-read genomic sequence analysis identified a third synonymous variant (c.660C>G, p.R220R) and demonstrated that all 3 variants were present in the same *SCN8A* allele. Importantly, both S217P and R220R are located within exon 5N, whereas T144S was in a neighboring exon not subject to alternative splicing. Because of the presumed developmentally regulated exclusion of exon 5N and uncertainty about which variant was pathogenic, we investigated the functional properties of the compound T144S/S217P/R220R genotype in neonatal Na_V_1.6N, and the single T144S variant in the Na_V_1.6A isoform (Figure 8).

Compared with the WT channel, the triple variant in Na_V_1.6N exhibited significantly smaller whole-cell currents, significantly hyperpolarized shifts in the voltage dependence of activation and inactivation, slower recovery from inactivation, and larger persistent and ramp currents (Figure 8, A–C, and Supplemental Data Set 2). By contrast, T144S expressed in the Na_V_1.6A isoform exhibited mostly WT-like properties, with the exception of slightly slower inactivation kinetics and modestly larger ramp current and window current (Figure 8, D and E, and Supplemental Data Set 3). Although only the triple variant channel had altered voltage dependence of inactivation, both variants exhibited slower time course of inactivation measured at 0 mV, which was greater for the compound variant expressed in Na_V_1.6N. We concluded that the triple variant channel in the neonatal splice isoform exhibits mixed dysfunctional properties that individually represented either loss of function (e.g., smaller current density, hyperpolarized voltage dependence of inactivation, slower recovery from inactivation) or gain of function (e.g., hyperpolarized activation voltage dependence, slower time course of fast inactivation, larger persistent and ramp currents) and is the likely pathogenic driver of the clinical phenotype. This also suggests that suppression of exon 5N incorporation into mature *SCN8A* mRNA transcripts may be a therapeutic strategy in this case.

## Discussion

In this study, we demonstrated the importance of considering alternative splicing when assessing disease-associated *SCN8A* variants, and validated 2 experimental strategies for accomplishing this task (creation of a neuronal cell line with low endogenous sodium currents and use of automated patch clamp recording). In combination, these approaches provide the means for greater physiological relevance and higher throughput that will help standardize efforts to determine the functional consequences of *SCN8A* variants, which is important for assessing variant pathogenicity, ascertaining molecular pathogenesis, and for screening therapeutic agents (21). In particular, automated patch clamp recording is increasingly used to assess the functional consequences of human ion channel variants at a scale difficult to achieve with traditional electrophysiological methods (28–38).

A focus of our study was on comparing the functional properties of WT and variant Na_V_1.6 expressed in 2 distinct splice isoforms of the channel (Na_V_1.6N and Na_V_1.6A). Human brain Na_V_ channel genes undergo developmentally regulated alternative splicing, including a well-characterized event involving 2 distinct versions of the fifth coding exon that encode portions of the first voltage-sensing domain (8). Transcriptome profiling of human brain indicates that Na_V_1.6N is the most abundant *SCN8A* transcript before birth, whereas Na_V_1.6A becomes the major splice isoform by around age 1 year (9), with a transition sometime during infancy, but there is interindividual variability in the timing of this event and it is not known how pathogenic *SCN8A* variants affect alternative splicing.

At the typical age of onset of neurological symptoms in *SCN8A*-related disorders (4–6 months), there is a mix of splice isoforms containing either exon 5A or 5N (9). Published studies on the functional consequences of *SCN8A* variants only use cDNA constructs representing rodent Na_V_1.6 (splice isoform unclear) (11–13) or the neonatal splice isoform of the human channel (14–19, 39). It is conceivable that pathogenic *SCN8A* variants disrupt the timing of alternative splicing in one direction or the other. Our study considered both splice isoforms to ensure we captured the most physiologically relevant molecular context, and determined that the functional properties for some variants are isoform dependent.

*SCN8A* exons 5N and 5A encode proteins that differ by 2 amino acids at positions 207 (5A: isoleucine; 5A: valine) and 212 (5N: asparagine; 5A: aspartate), and these differences are sufficient to affect the functional properties of the respective channel splice isoforms (Figure 2 and Supplemental Data Set 1). The neurophysiological consequences of this alternative splicing event for *SCN8A* in vivo are unknown, but the analogous splicing event in mouse Na_V_1.2 correlates with greater cortical pyramidal neuron excitability beginning after postnatal day 3 that persists into adulthood (40) and a computational model of a human cortical pyramidal neuron exhibits higher firing frequency when Na_V_1.2A is incorporated (41). Based on our electrophysiological observations, Na_V_1.6N exhibits slightly depolarized voltage dependence of both activation and inactivation that have the potential of affecting neuronal excitability. Prior studies suggested that shifts in Na_V_1.6 activation voltage dependence have a greater impact on neuronal firing than similar shifts in the voltage dependence of inactivation (16), and we could speculate that Na_V_1.6N expression renders immature neurons less excitable.

Because we considered the molecular context for *SCN8A* variants to be relevant to understanding their functional consequences, we investigated the functional properties of variants located exclusively in either exon 5A or 5N using the relevant splice isoform. One variant (R223G) was previously studied in a TTX-resistant version of human Na_V_1.6N expressed in ND7/23 cells (20), and findings from that study differ from our data on human Na_V_1.6A-R223G expressed in ND7/LoNav cells (20). Specifically, the previous study demonstrated hyperpolarized activation voltage dependence and faster recovery from inactivation, whereas our data indicated WT-like activation voltage dependence, and slower recovery from inactivation. Two other variants (R850Q and G1475R) previously studied in TTX-resistant Na_V_1.6N also exhibited functional differences with our study using the human Na_V_1.6A isoform. The previous work on R850Q highlighted large persistent current and hyperpolarized voltage dependence of activation (17), which we also observed, but our findings differed in the impact on inactivation voltage dependence and recovery from inactivation. Similarly, prior work on G1475R in human TTX-resistant Na_V_1.6N (16, 18) differed from our work with Na_V_1.6A in current density, persistent current, and ramp current. A recent study of G1625R performed in mouse Neuro-2a cells using human TTX-resistant Na_V_1.6N reported qualitatively similar, but quantitatively different, findings compared with our results (39). Not all variants, including variants N1768D and G1625R, exhibit isoform-dependent properties. A summary of differences between our study and previously reported work is presented in Supplemental Table 3.

The value of studying variants in the correct molecular context was also illustrated by our investigation of a complex genotype with 3 in-phase variants of uncertain significance discovered in an infant with severe DEE. This work allowed us to demonstrate which genotype was most likely to be pathogenic, and provided data supporting the potential therapeutic value of induced exon 5 splice switching (42).

In addition to considering the relevant splice isoform, we also developed a cellular platform for investigating *SCN8A* variants that exploits a neuronal cell environment without requiring a pharmacological intervention to isolate human Na_V_1.6 current. We developed ND7-LoNav cells by genetically inactivating endogenous rodent Na_V_1.7, resulting in a neuron-derived cell line without appreciable background sodium current. While this cell line has value for studying brain Na_V_ channel variants in vitro, it is important to point out that these cells are not human and were not derived from central neurons. Separate studies in native neurons may have additional value in relating the functional effects of variants to neuronal physiology.

Use of LoNav cells obviates the need for a second site mutation to render human Na_V_1.6 TTX resistant (14, 16–19). Our approach avoids the potential confounding effects of TTX-resistance mutations at amino acid position 371 (Y371C and Y371S), which we demonstrated have significant functional differences from WT channels in either Na_V_1.6N or Na_V_1.6A. Other studies have shown that a corresponding TTX-resistance mutation in Na_V_1.3 (Y384S) also exhibits dysfunctional properties (43). We raise concern that some prior findings may be confounded by extra pore domain mutations.

One goal of determining the functional consequences of *SCN8A* variants is to aid in building genotype-phenotype correlations. Such correlations are valuable for understanding differences in pathophysiological mechanisms and may help guide pharmacological therapy such as the use of Na_V_ channel–blocking antiseizure medications, which would be most useful in the setting of gain-of-function variants. The majority of variants we studied exhibited multiple functional disturbances that individually are consistent with either gain or loss of function, but the net effect of these effects can be challenging to determine without additional experimental work. For some variants such as N1768D, enhanced persistent current combined with depolarized voltage dependence of inactivation are likely drivers of elevated seizure susceptibility. However, even this widely accepted gain-of-function variant exhibits other functional properties that are consistent with loss of function, including depolarized voltage dependence of activation (Figure 4), slower recovery from inactivation (Supplemental Figure 6), and a greater tendency for frequency-dependent loss of activity (Supplemental Figure 7). Of note, we did not interrogate resurgent current in our study, but previous studies suggested that this biophysical feature can be affected by certain *SCN8A* variants (14, 17). Complementary experimental approaches with computational simulation of neuronal action potentials (44) or dynamic action potential clamp (45, 46) may be valuable next steps.

In summary, we standardized an approach for evaluating the functional consequences of *SCN8A* variants that exploits the higher throughput achievable with automated patch clamp recording and obviates the need for second-site TTX-resistance mutations through use of a neuronal cell line with low levels of endogenous Na_V_ current. Our findings demonstrate that developmentally regulated alternative splicing of exon 5 influences variant function and emphasize the importance of studying variants in physiologically relevant splice isoforms.

## Methods

### Sex as a biological variable.

Our study indirectly involved deidentified human participants who have a rare disorder. Sex was not declared for all participants. We were not powered to address sex as a biological variable.

### Long-read SCN8A genomic sequencing.

Cheek swab genomic DNA was obtained from a proband carrying 2 de novo *SCN8A* missense variants and from both parents. To determine the phase of the 2 variants in the proband, long-range genomic PCR was performed under dilute conditions with long extension times and Phusion High Fidelity Polymerase (Thermo Fisher Scientific) using primers targeting an amplicon of approximately 2.6 kb encompassing both variants (forward: CTCTTCTGTGCTTCACCTTTCTCTAGC; reverse: CCTATCCCAACACCTAACACCAACC). Samples were analyzed for quantity and quality using UV-Vis spectrometry and Femto Pulse (Agilent) pulsed-field capillary electrophoresis, followed by standard SMRTbell PacBio (Pacific Biosciences) library preparation. Library quality control was done using Qubit fluorometric analysis (Thermo Fisher Scientific).

Long-read sequencing of the 3 libraries was performed using a single flow cell on a PacBio Sequel v3r9 (Pacific Biosciences) at the Massachusetts Institute of Technology Biomicro Center using PacBio Circular Consensus Sequencing to improve base-calling accuracy. Sequence reads from the proband sample were first filtered for lengths in the 2500–2700 bp range. Sequences were then filtered for the presence of four 21-bp “anchor” segments (A, B, C, and D) arranged AV_1_B to CV_2_D. Anchor segments were defined as 21-bp reference genomic sequences located immediately upstream or downstream of each variant (V_1_, V_2_), with no filter on the identity of the base at the V_1_ or V_2_ position. After discovery of a third synonymous variant (V_3_) described below, the filter for the D anchor was adjusted to allow any base at the location of V_3_ in this anchor. This filtering yielded 73,522 sequences. Sequences were scored for the presence of the variant (V) or reference (R) allele at the V_1_ or V_2_ position, yielding 46% V/V, 40% R/R, 7% V/R, and 7% R/V sequences, indicating a relatively low representation of chimeric products, and indicating that the 2 variants are in *cis*. Similar analyses of the parental samples yielded approximately 99% reference allele at both variant positions, confirming that both variants arose de novo in the proband. Visual inspection of multiple sequence alignments of proband amplicon sequences also revealed the presence of V_3_ — g.52082587C>G (chr12, hg19) — located 11 bp downstream of V_2_. This variant was observed in more than 99% of proband sequences containing V_2_ and in virtually none of the sequences containing the reference allele at this position, confirming that it is in *cis* relative to V_2_ (and V_1_ by inference). The V_3_ variant was not observed in the parental sequences, confirming that it also arose de novo in the proband.

### Plasmids and mutagenesis.

Plasmids encoding human Na_V_1.6 splice isoforms annotated by NCBI as variant 1 (NM_014191; neonatally expressed, Na_V_1.6N) and variant 3 (NM_001177984; adult expressed, Na_V_1.6A) were rendered stable in bacteria by inserting small introns at the exon 14-15 and 22-23 junctions, as described previously (47). Plasmids included an IRES2 element followed by the reading frame for the red fluorescent protein mScarlet to enable determination of transfection efficiency. Both plasmids are available from AddGene (Na_V_1.6N, 162280; Na_V_1.6A, 209411). *SCN8A* variants were introduced into WT Na_V_1.6A or Na_V_1.6N using PCR mutagenesis with Q5 Hot Start High-Fidelity 2× Master Mix (New England Biolabs), as described previously (47). Primers were designed for each mutation using custom software to have a minimum 5′ overlap of 20 bp and a predicted melting temperature (Tm) of 60°C (Supplemental Table 4). All recombinant plasmids were sequenced in their entirety using nanopore-based sequencing (Primordium Labs) to confirm the presence of the desired modifications and the absence of inadvertent mutations.

### Cell culture.

ND7/23 (Sigma-Aldrich) and ND7/LoNav (see below) cells were grown at 37°C with 5% CO_2_ in Dulbecco’s modified Eagle’s medium (DMEM) supplemented with 10% fetal bovine serum (ATLANTA Biologicals), 2 mM L-glutamine, 50 U/mL penicillin, and 50 μg/mL streptomycin. Unless otherwise stated, all tissue culture media were obtained from Thermo Fisher Scientific.

### Generation of ND7/LoNav cells.

Transient knockdown of Na_V_1.7 mRNA was done using short interfering RNA (siRNA) targeting both mouse and rat channel transcripts (4390771, ID: s134909; Thermo Fisher Scientific/Ambion). Permanent knockout of endogenous Na_V_1.7 in ND7/23 cells was achieved using CRISPR/Cas9 genome editing with plasmid pD1301-AD (pCMV-Cas9-2A-GFP) encoding a guide RNA (gRNA) sequence (GTTACTGCTGCGCCGCTCCC) targeting both mouse *Scn9a* (GRCM39, chr2:66370779–66370798) and rat *Scn9a* (mRatBN7.2, chr3:51197912–51197931). Genome-editing vectors were synthesized by ATUM. ND7/23 cells were transfected with FuGENE 6 Transfection Reagent (Promega) according to the manufacturer’s instructions. Two days after transfection, cells were flow sorted and green fluorescent cells with the top 50% intensity were isolated for single-clone selection. Sequencing of PCR amplicons encompassing the edited regions that were subcloned into the TOPO-TA vector (Invitrogen) revealed a subset with in-frame deletions in mouse *Scn9a*. An additional round of CRISPR/Cas9 genome editing was performed on 1 clonal cell line to further disrupt the coding region of endogenous *Scn9a* using recombinant pX458 plasmid (pSpCas9-2A-GFP; Addgene, 48138) and a gRNA targeting the in-frame deletion (*Scn9a*, CTATTTGTACCCCATAAG). After transfection and flow sorting of green fluorescent cells, clonal lines with the lowest inward current amplitude determined by whole-cell automated patch clamp recording were selected.

### Immunoblotting.

Mouse and rat Na_V_1.6 and Na_V_1.7 were detected using anti-Na_V_1.6 antibody (1:100 dilution; ASC-009, Alomone Labs) and anti-Na_V_1.7 antibody (1:200 dilution; 75-103, UC Davis/NIH NeuroMab Facility), respectively. Transferrin receptor (loading control) was detected with mouse anti–human transferrin receptor antibody (1:500; 136800, Invitrogen). Protein samples (50 μg) were electrophoresed in 7.5% polyacrylamide Mini-PROTEAN TGX gels (4561023, Bio-Rad), electrotransferred to Immobilon-P PVDF membranes (pore size 0.45 μm, IPFL00010, MilliporeSigma), and blocked in 5% bovine serum albumin for 1 hour. Sodium channels were detected by first incubating with anti-Na_V_1.6 or -Na_V_1.7 primary antibodies followed by incubation with IRDye 800CW goat anti-mouse antibody (1:10,000; 926-322100, LI-COR Biosciences). Transferrin receptor was detected with primary antibody followed by incubation with IRDye 680RD goat anti-mouse antibody (1:10,000; 926-68070, LI-COR Biosciences). Protein bands were imaged with the Odyssey CLx Imaging System (LI-COR Biosciences).

### Transfections.

WT and variant human Na_V_1.6A and Na_V_1.6N plasmids were transiently expressed in ND7/LoNav cells using the STX system (MaxCyte Inc.). ND7/LoNav cells were seeded at 1.1 × 10^6^ cells/mL in 100-mm tissue culture dishes and grown to 65%–75% density, and then harvested using TrpLE Express (12605010, Thermo Fisher Scientific/GIBCO). A 500 μL aliquot of cell suspension was used to determine cell number and viability using an automated cell counter (ViCell, Beckman Coulter). Remaining cells were collected by gentle centrifugation (160*g*, 4 minutes), washed with 5 mL electroporation buffer (EBR100, MaxCyte Inc.), and resuspended in electroporation buffer at a density of 1 × 10^8^ viable cells/mL. Each electroporation was performed using 100 μL of cell suspension.

ND7/LoNav cells were electroporated with 50 μg of WT or variant Na_V_1.6 cDNA. The DNA-cell suspension mix was transferred to an OC-100 processing assembly (MaxCyte Inc.) and electroporated using the preset Optimization 4 protocol. Immediately after electroporation, 10 μL of DNase I (Sigma-Aldrich) was added to the DNA-cell suspension. Cell-DNA-DNase mixtures were transferred to 6-well tissue culture plates and incubated for 30 minutes at 37°C in 5% CO_2_. Following incubation, cells were gently resuspended in culture media, transferred to 100-mm tissue culture dishes, and grown for 48 hours at 37°C in 5% CO_2_. Following incubation, cells were harvested, counted, transfection efficiency determined by flow cytometry (see below), and then frozen in 1 mL aliquots at 1.8 × 10^6^ viable cells/mL in liquid nitrogen until used in experiments. Mean transfection efficiency was 82%, and only batches of cells exhibiting an efficiency of greater than 65% were used in experiments.

Transfection efficiency was evaluated by flow cytometry using a FACSCanto (BD Biosciences) located in the Northwestern University Interdepartmental Immunobiology Flow Cytometry Core Facility. Forward scatter, side scatter, and red fluorescence were measured using a 488 nm laser.

### Manual patch clamp recording.

Currents were recorded at room temperature in the whole-cell configuration while acquired at 20 kHz and filtered at 5 kHz. Bath solution contained (in mM): 145 NaCl, 4 KCl, 1.8 CaCl_2_, 1 MgCl_2_, 10 HEPES, pH 7.35, and 310 mOsm/kg. The composition of the pipette solution was (in mM): 10 NaF, 110 CsF, 20 CsCl, 2 EGTA, 10 HEPES, pH 7.35, 310 mOsm/kg. Whole-cell patch pipettes were pulled from thin-wall borosilicate glass (Warner Instruments, LLC) with a multistage P-97 Flaming-Brown micropipette puller (Sutter Instruments Co.) and fire-polished with a Micro Forge MF 830 (Narashige International). Pipette resistance was approximately 2 MΩ.

### Cell preparation for automated electrophysiology.

Electroporated cells were thawed the day before experiments, plated in 60-mm tissue culture dishes, and incubated for 18–24 hours at 37°C in 5% CO_2_. Prior to experiments, cells were detached using TrpLE Express, resuspended in cell culture media, and counted. Cells were centrifuged at 100*g* for 2 minutes and then resuspended at 180,000/mL with external solution (see below) and allowed to recover 45 minutes at 15°C while shaking 200 rpm on a rotating platform.

### Automated patch clamp.

Automated planar patch clamp recording was performed using a SyncroPatch 768 PE (Nanion Technologies), as previously described (28). External solution contained (in mM): 140 NaCl, 4 KCl, 2.0 CaCl_2_, 1 MgCl_2_, 10 HEPES, 5 glucose, pH 7.4. The composition of the internal solution was (in mM): 10 NaF, 110 CsF, 10 CsCl, 20 EGTA, 10 HEPES, pH 7.2. Whole-cell currents were acquired at 10 kHz and filtered at 3 kHz. The access resistance and apparent membrane capacitance were determined. Series resistance was compensated 90%, whereas leak and capacitance artifacts were subtracted using the P/4 method.

Data were analyzed and plotted using a combination of DataController384 version 1.8 (Nanion Technologies), Excel (Microsoft Office 2013, Microsoft), SigmaPlot 2000 (Systat Software, Inc.), and Prism 8 (GraphPad Software), as previously described (28). Whole-cell currents were measured from a holding potential of –120 mV. Whole-cell conductance (G_Na_) was calculated as G_Na_ = *I*/(*V* − *E_rev_*), where *I* is the measured peak current, *V* is the step potential, and *E_rev_* is the calculated sodium reversal potential. G_Na_ at each voltage step was normalized to the maximal conductance between −80 mV and 20 mV. To calculate voltage dependence of activation and inactivation, data were plotted against voltage and fitted with Boltzmann functions. Time-dependent recovery from inactivation was evaluated by fitting peak current recovery with a 2-exponential function. Time-dependent entry into inactivation was evaluated by fitting current decay at 0 mV with a single exponential function. Number of cells (*n*) is given in the figure legends. Persistent current was measured as the ratio of peak ramp current to the peak current measured at 0 mV during the activation protocol, and charge movement was calculated as the integral of the ramp current divided by the current measured during the activation protocol. Window current was calculated by integrating the area under the intersection between the Boltzmann fits for voltage dependence of activation and inactivation using a custom MatLab script (48).

### Statistics.

Data from individual cells expressing variant Na_V_1.6 were compared to the average of the isoform-matched WT Na_V_1.6 run in parallel using a 2-tailed *t* test. Statistical significance was established at a *P* value of 0.01 or less, which represents a Bonferroni’s correction for multiple testing in experiments comparing 4 variants to WT channels. One-way ANOVA was used to compare functional properties of WT Na_V_1.6 splice isoforms and nontransfected cells, with statistical significance set at a *P* value of 0.01 or less. Unless otherwise noted, data are presented as mean ± 95% confidence intervals (CIs).

### Study approval.

For unpublished *SCN8A* variants, we obtained informed consent from parents to allow publication of brief deidentified clinical descriptions and genetic information using a method approved by the Children’s Hospital of Philadelphia Institutional Review Board.

### Data availability.

The authors confirm that the data supporting the findings of this study are available within the article and the supplemental material, including Supplemental Data Sets and the Supporting Data Values file.

## Author contributions

CGV, TVA, JMD, NFG, MJO, CBB, and CHT performed experimental work and analyzed data. IH acquired informed consent. ALG acquired funding and supervised the work. The manuscript was written primarily by CGV and ALG, with input from all co-authors. All authors reviewed and approved the final version of the manuscript prior to submission.

## Supplementary Material

Supplemental data

Supplemental data set 1

Supplemental data set 2

Supplemental data set 3

Unedited blot and gel images

Supporting data values

## Version 1 05/21/2024

In-Press Preview

## Version 2 06/24/2024

Electronic publication
