# Supplementary material for: Molecular and cellular context influences SCN8A variant function
Source: JCI Insight. 2024 May 21;9(12):e177530. doi: 10.1172/jci.insight.177530 (PMC11383174; doi:10.1172/jci.insight.177530)
Supplement: Supplemental data [file jciinsight-9-177530-s100.pdf]

Molecular and Cellular Context Influences *SCN8A* Variant Function

Carlos G. Vanoye, Tatiana V. Abramova, Jean-Marc Dekeyser, Nora F. Ghabra, Madeleine J. Oudin, Christopher B. Burge, Ingo Helbig, Christopher H. Thompson, and Alfred L. George Jr.

SUPPLEMENTAL INFORMATION

Supplemental Figures

Fig. S1. Western blot of $Na_V1.6$ in ND7/23 cells

Fig. S2. Location of $Na_V1.6$ variants analyzed in this study.

Fig. S3. Whole-cell currents for variants expressed in $Na_V1.6N$.

Fig. S4. Whole-cell currents for variants expressed in $Na_V1.6A$.

Fig. S5. Window current determined for $Na_V1.6$ variants.

Fig. S6. Recovery from inactivation determined for $Na_V1.6$ variants.

Fig. S7. Use-dependent rundown of $Na_V1.6$ variants.

Fig. S8. Comparison of functional properties among SCN8A variants.

Fig. S9. Heat map illustrating functional properties of $Na_V1.6$ variants.

Supplemental Tables

Table S1. Clinical phenotypes associated with *SCN8A* variants in this study.

Table S2. Summary of major functional properties of *SCN8A* variants.

Table S3. Comparison of reported functional properties of *SCN8A* variants.

Table S4. Mutagenic primer sequences for *SCN8A* variants.

Supplemental References

Supplemental Datasets (separate files)

Dataset S1. Biophysical properties of WT $Na_V1.6A$ and WT $Na_V1.6N$ (Excel file).

Dataset S2. Biophysical properties of variants studied in $Na_V1.6N$ (Excel file).

Dataset S3. Biophysical properties of variants studied in $Na_V1.6A$ (Excel file).

SUPPLEMENTAL FIGURES

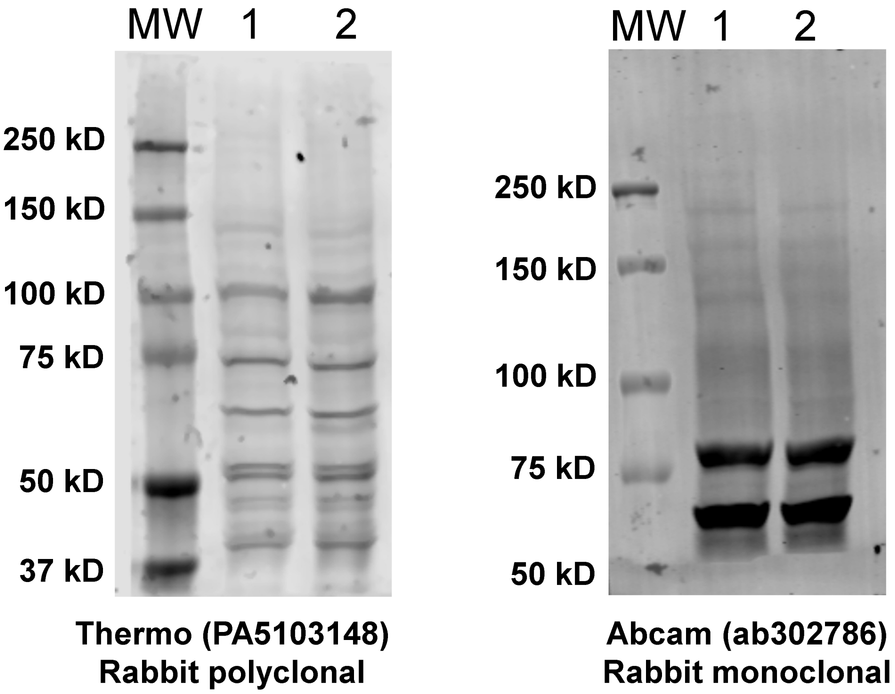

Fig. S1. Western blot of Na_V1.6 in ND7/23 and ND7/LoNav cells.
Western blots of whole cell lysates from (1) ND7/23 or (2) ND7/LoNav cells probed with two different antibodies. First lanes are molecular weight (MW) markers. Blots were exposed to enhance detection of protein bands in the range of ~250 kDa, which would be consistent with Na_V1.6.

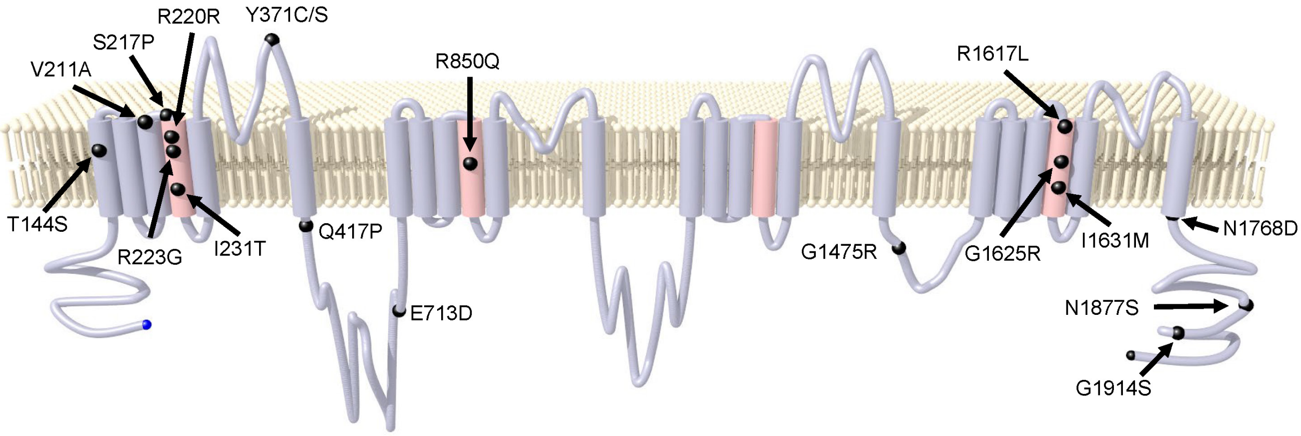

Fig. S2. Location of $Na_V1.6$ variants analyzed in this study.
Simplified transmembrane topology of $Na_V1.6$ with locations of variants studied.

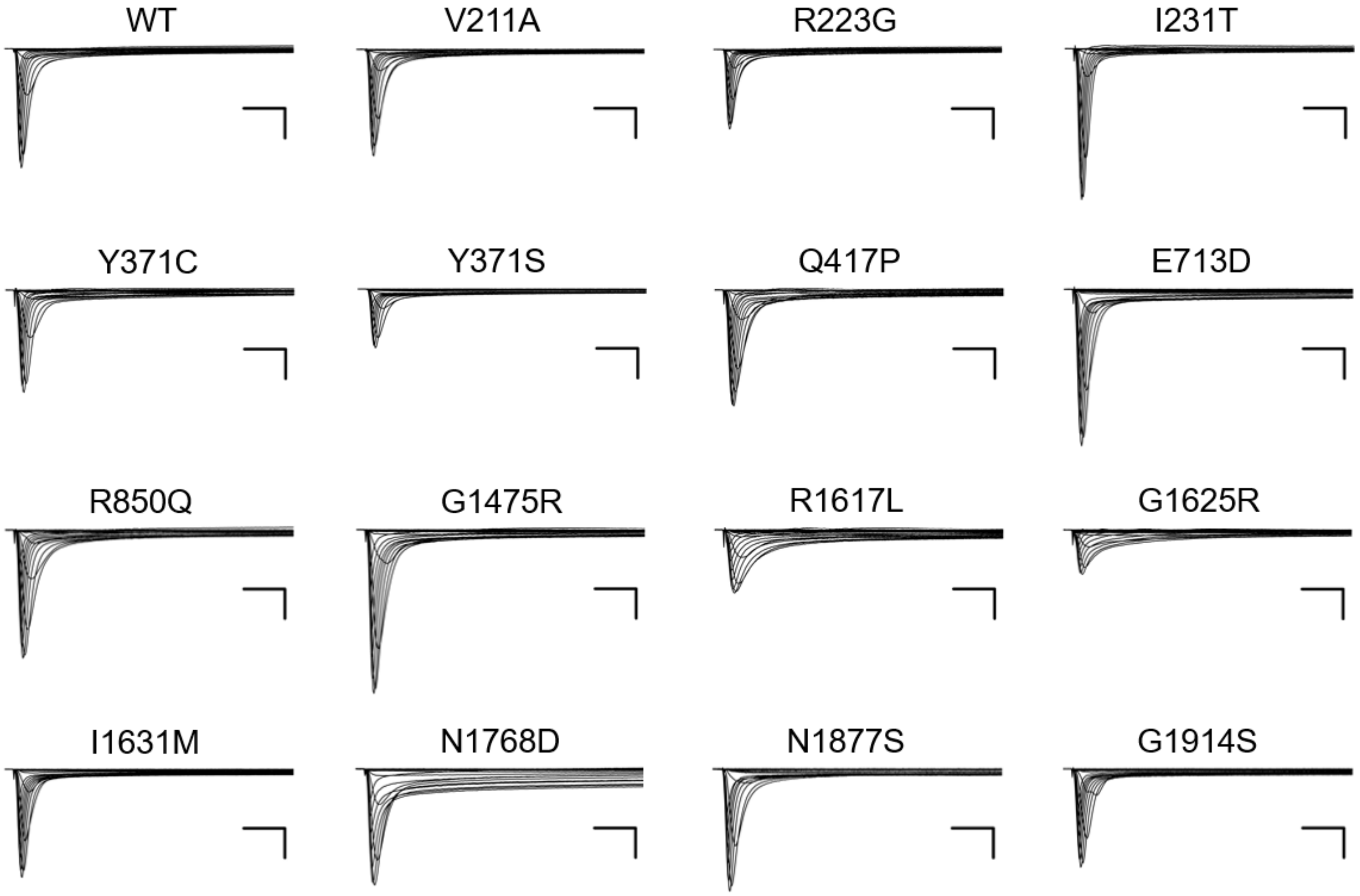

Fig. S3. Whole-cell currents for variants expressed in $Na_V1.6A$.

Averaged whole-cell currents recorded from ND7/LoNav cells transfected with $Na_V1.6$ variants and normalized to the WT channel peak current recorded in parallel (n = 27-64 per variant). Scale bars are 5 ms (horizontal) and 25% of WT channel current density (vertical).

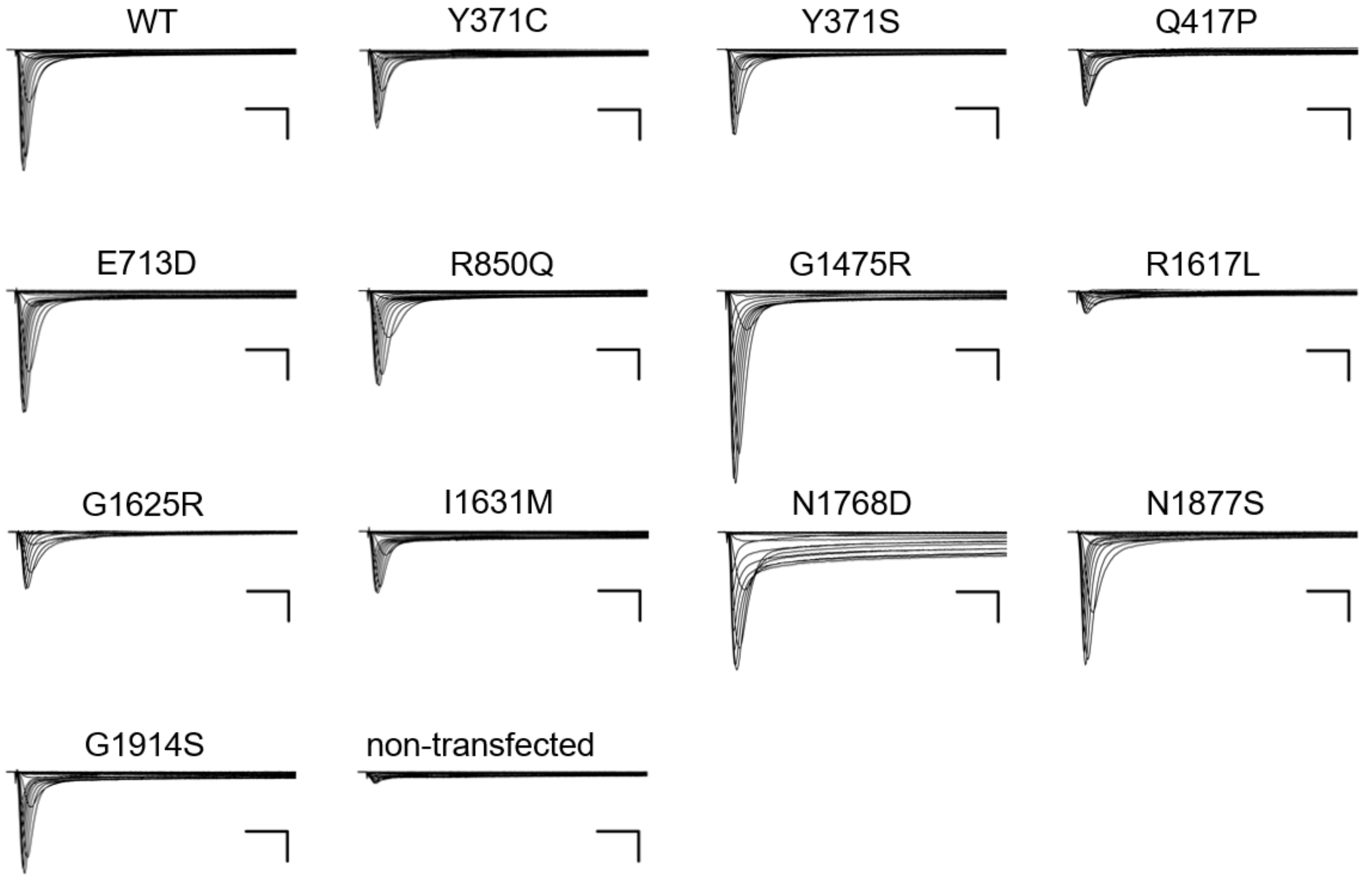

Fig. S4. Whole-cell currents for variants expressed in $Na_V1.6N$.
Averaged whole-cell currents recorded from ND7/LoNav cells transfected with $Na_V1.6$ variants and normalized to the WT channel peak current recorded in parallel (n = 25-67 per variant). Scale bars are 5 ms (horizontal) and 25% of WT channel current density (vertical).

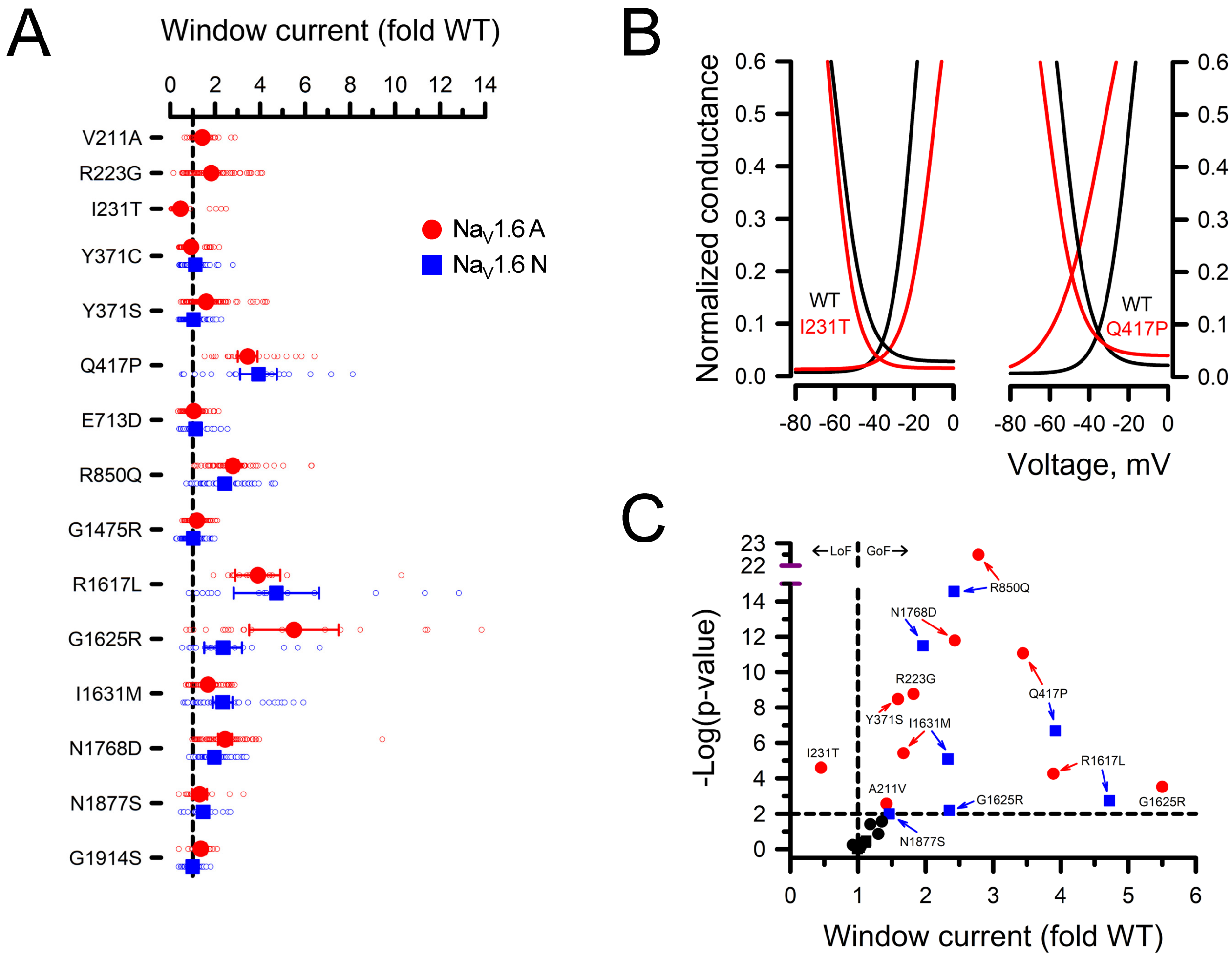

Fig. S5. Window current determined for Na$_V$1.6 variants.

(A) Average deviation from WT Na$_V$1.6 for window current area. All individual data points are plotted as open symbols and mean values are shown as larger filled symbols (n = 14-69). Error bars represent 95% CI. Data from Na$_V$1.6A or Na$_V$1.6N are indicated as red or blue symbols, respectively. Values to the right or left of the vertical dashed line (average normalized WT value) indicate larger (gain-of-function) or smaller (loss of function) window current, respectively. (B) Averaged Boltzmann fit lines for activation and steady-state inactivation curves of representative variants with smaller (I231T) or larger (Q417P) window current. (C) Volcano plot of mean values highlighting variants significantly different (P<0.01, horizontal dashed line) from WT. Symbols to the right of the vertical dashed line represent larger window current (gain-of-function). Only one variant (I231T) exhibited significantly smaller window current. Black symbols represent variants with no significant difference from WT. Quantitative data with statistical comparisons are provided in Supplemental Dataset S2 (Na$_V$1.6N) and Supplemental Dataset S3 (Na$_V$1.6A).

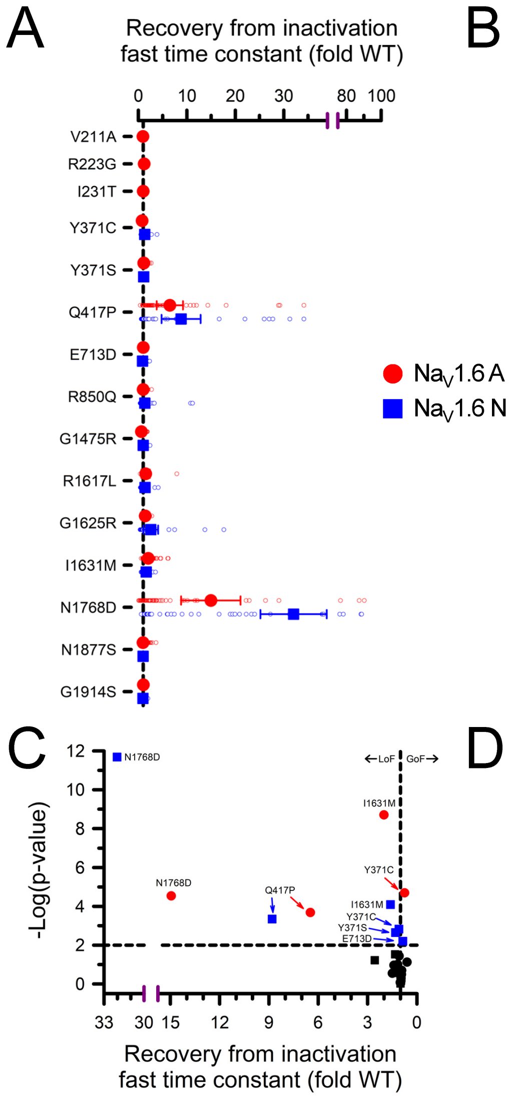

Fig. S6. Recovery from inactivation determined for Na_V1.6 variants.

(A,B) Averaged time constants for recovery from inactivation displayed as fold-difference from WT channels recorded in parallel. Time constants (fast component plotted in panel A, slower component plotted in panel B) were determined by fitting the time course of recovery from inactivation to a double exponential function. All individual data points are plotted as open symbols and mean values are shown as larger filled symbols (n = 12-105 per variant). Error bars represent 95% confidence intervals. Data from Na_V1.6A or Na_V1.6N are indicated as red or blue symbols, respectively. Values to the right or left of the vertical dashed line (normalized WT value) represent larger (slower recovery) or smaller (faster recovery) time constants, respectively. (C,D) Volcano plots highlighting variants with significantly different (P <0.01, horizontal dotted line) fast component (C) and slower component (D) time constants of recovery from inactivation. Symbols to the left of the vertical dotted line denote slower recovery time course (loss-of-function), while symbols to right indicate faster recovery time course (gain-of-function). Black symbols represent variants with no significant difference from WT. Quantitative data with statistical comparisons are provided in Supplemental Dataset S2 (Na_V1.6N) and Supplemental Dataset S3 (Na_V1.6A).

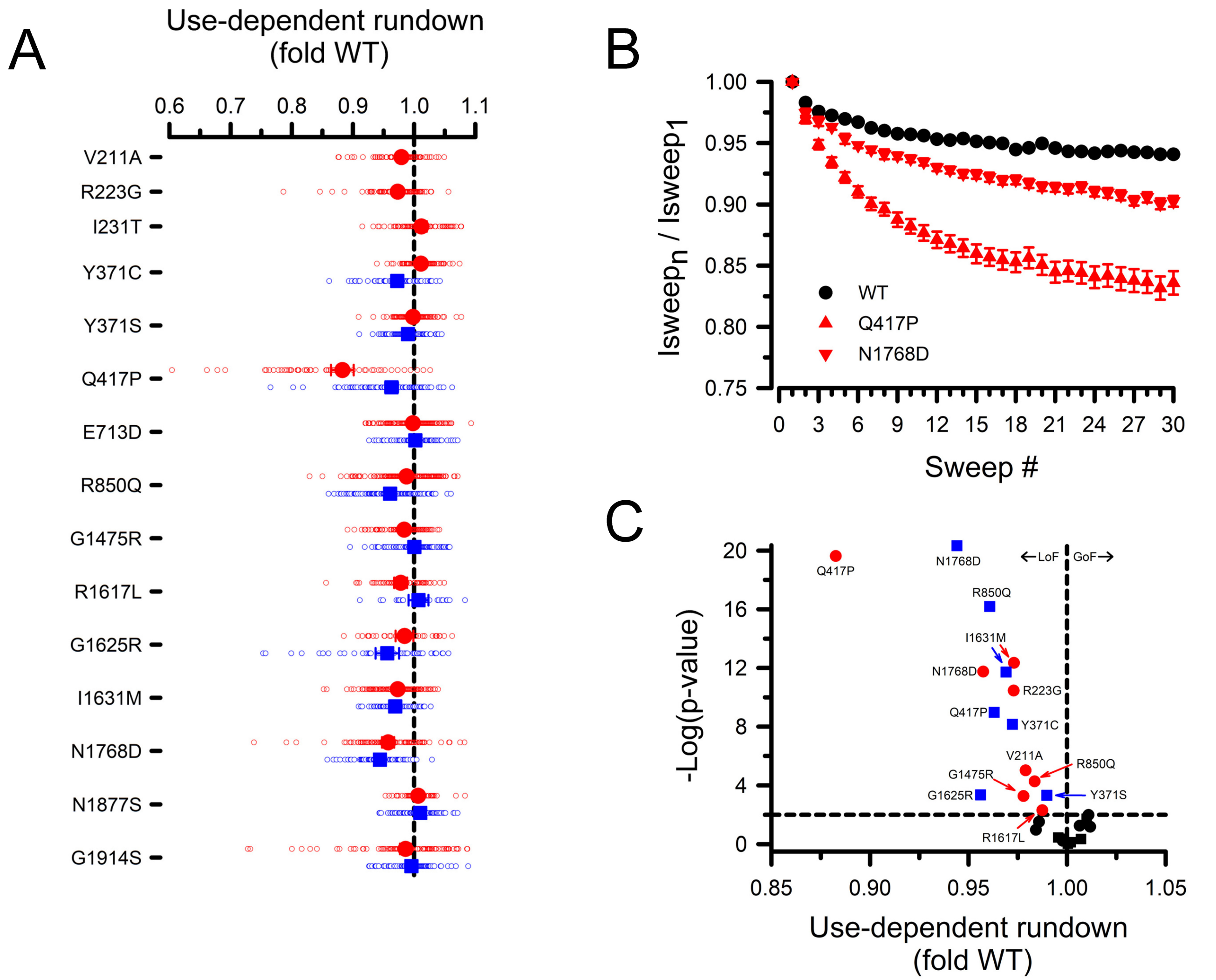

Fig. S7. Use-dependent rundown of Na$_V$1.6 variants.

(A) Averaged use-dependent channel rundown at 20 Hz measured for Na$_V$1.6 variants displayed as fold-difference from WT channels recorded in parallel. All individual data points are plotted as open symbols and mean values are shown as larger filled symbols (n = 26-153 per variant). Error bars represent 95% confidence intervals. Data from Na$_V$1.6A or Na$_V$1.6N are indicated as red or blue symbols, respectively. Values to the left of the vertical dashed line (normalized WT value) represent greater rundown than WT. No variants exhibited lesser degree of rundown than WT. (B) Averaged currents normalized to first sweep amplitude measured for 30 sweeps at 20 Hz for select variants expressed in Na$_V$1.6A. (C) Volcano plot of mean values highlighting variants with significantly different (P<0.01, horizontal dashed line) use-dependent rundown from WT. Symbols to the left of the vertical dashed line represent greater rundown (loss-of-function and there were no variants with lesser rundown. Black symbols represent variants with no significant difference from WT. Quantitative data with statistical comparisons are provided in Supplemental Dataset S2 (Na$_V$1.6N) and Supplemental Dataset S3 (Na$_V$1.6A).

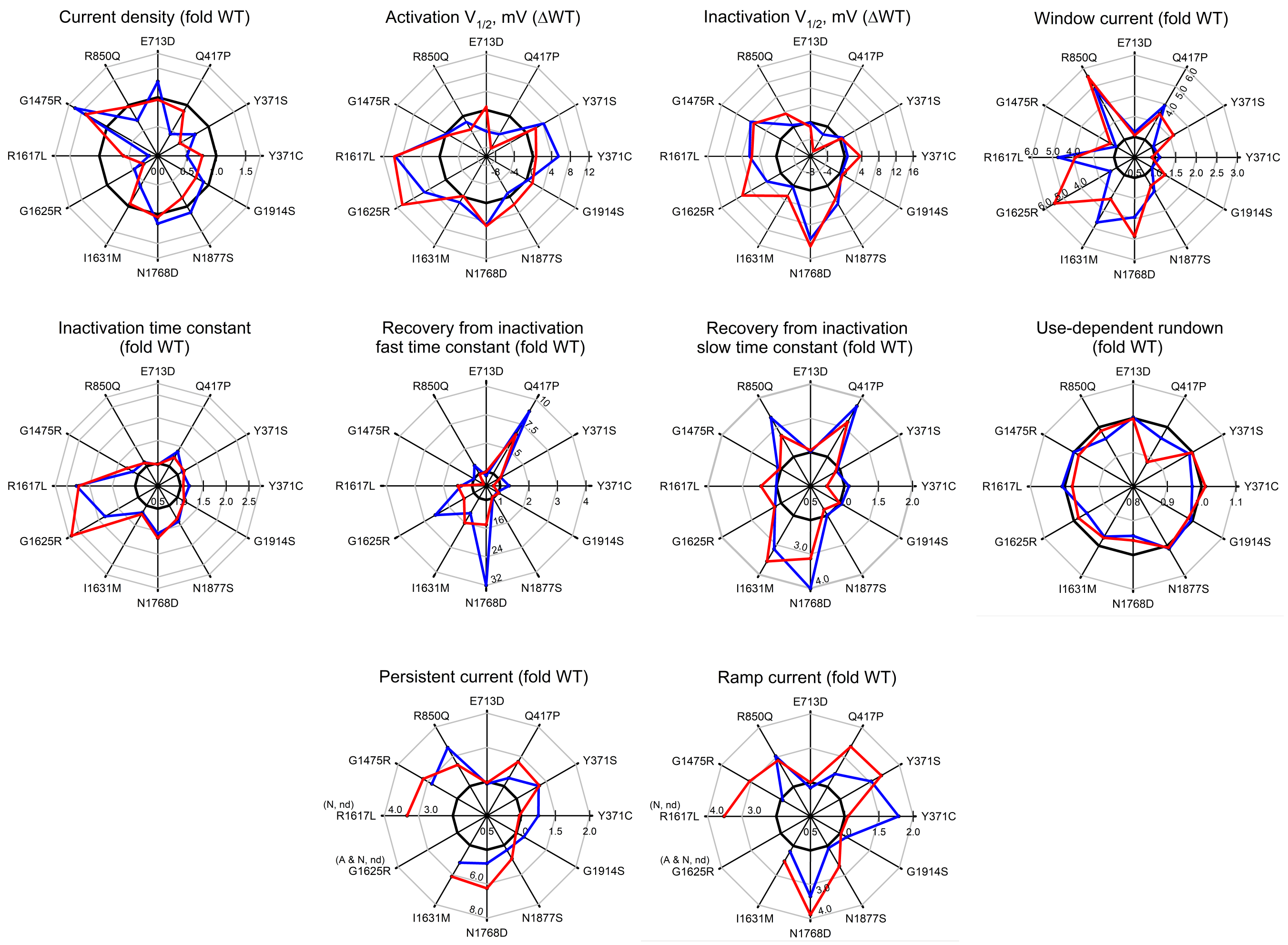

Fig. S8. Comparison of functional properties among SCN8A variants.

Radar plots depicting biophysical properties among variants compared to the WT channel. Individual radar plots represent different properties and each individual variant is represented as points (mean values normalized to WT) along each spoke. Red lines connecting each point represent data from $Na_V1.6A$, blue lines represent data from $Na_V1.6N$, and black lines indicate isoform-matched WT values. The scale indicating the magnitude of difference for each biophysical property is shown within the radar plot on the Y371C spoke, except for individual variants and specific properties (e.g., Window Current for Q417P and R1617L; Persistent Current for R1617L and N1768D; and Ramp Current for R1617L and N1768D). Persistent Current and Ramp Current were not determined (nd) for some variants. Quantitative data with statistical comparisons are provided in Supplemental Dataset S2 ($Na_V1.6N$) and Supplemental Dataset S3 ($Na_V1.6A$).

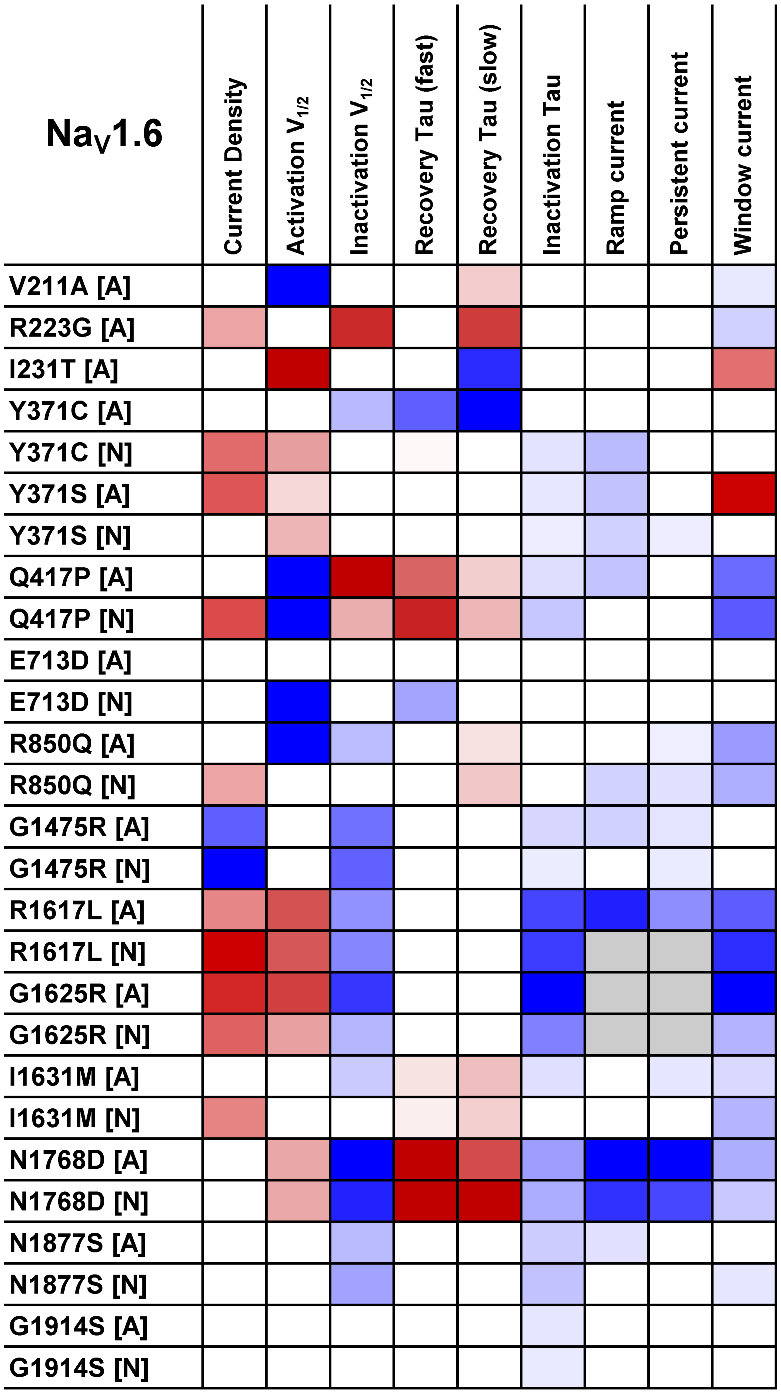

Fig. S9. Heat map illustrating functional properties of $Na_V1.6$ variants.

Summary of individual functional properties for variants expressed in either $Na_V1.6A$ or $Na_V1.6N$ are illustrated as a heat map. Properties with gain-of-function are shaded as blue, and loss-of-function as red. Gray shaded boxes are properties that were not determined, and uncolored boxes indicate WT-like values. Only properties that reached the threshold for statistical significance (P<0.01) are color-coded. The intensity of shading reflects the degree of difference with WT.

Table S1 – Clinical phenotypes associated with *SCN8A* variants in this study.

Nucleotide	Protein	Recurrent	Phenotype	Age at onset	Supplemental Reference(s)
c.632T>C	V211A	N	Epileptic encephalopathy	3 m	Exon 5A; (1)<br>Exon 5N: (2)
c.667A>G	R223G	N	West syndrome	4-6 m	Exon 5A: (1)<br>Exon 5N: (3)<br>Unclear: (4)
c.692T>C	I231T	N	Epileptic encephalopathy	7-8 m	Exon 5N: (1)
c.1250A>C	Q417P	Y	Intractable infantile spasms, global developmental delay	< 12 m	This study and (2)
c.2139A>C	E713D	N	Variant of uncertain significance	Unknown	ClinVar ID 838164
c.2549G>A	R850Q	Y	Epileptic encephalopathy	2 m	(5-8)
c.4423G>A	G1475R	Y	Epileptic encephalopathy, intermediate severity	1 – 11 m	(6-13)
c.4850G>T	R1617L	Y	Early-onset epilepsy	3-6 m	(5, 7, 8, 10, 12, 14, 15)
c.4893C>G	I1631M	N	Post-vaccination generalized seizures, later-onset partial epilepsy, mild intellectual disability	5 m	This study
c.4873G>A	G1625R	Y	Intellectual disability, developmental delay	unknown	(16, 17)
c.5302A>G	N1768D	N	Epileptic encephalopathy, SUDEP	6 m	(18)
c.5630A>G	N1877S	Y	Self-limited infantile epilepsy	5 m	(19, 20)
c.5740G>A	G1914S	N	Variant of uncertain significance	unknown	ClinVar ID 2500085

Table S2 – Summary of major functional effects of *SCN8A* variants in this study (differences between splice isoforms are in bold).

Variant	Functional effects in Na_V1.6A	Functional effects in Na_V1.6N
V211A	GOF: hyperpolarized activation $V_{1/2}$,<br>larger window current<br>LOF: slower recovery from inactivation	Not studied (variant restricted to exon 5A)
R223G	GOF: larger window current<br>LOF: smaller peak current density,<br>hyperpolarized inactivation $V_{1/2}$,<br>slower recovery from inactivation	Not studied (variant restricted to exon 5A)
I231T	GOF: faster recovery from inactivation<br>LOF: depolarized activation $V_{1/2}$	Not studied (variant restricted to exon 5A)
Y371C	GOF: **depolarized inactivation $V_{1/2}$**,<br>**faster recovery from inactivation**	GOF: **slower inactivation time course**,<br>**larger ramp current**<br>LOF: **smaller peak current density**,<br>**depolarized activation $V_{1/2}$**,<br>**slower recovery from inactivation**
Y371S	GOF: slower inactivation time course,<br>larger ramp current<br>LOF: smaller peak current density,<br>depolarized activation $V_{1/2}$	GOF: slower inactivation time course,<br>larger ramp current,<br>**larger persistent current**<br>LOF: smaller peak current density,<br>depolarized activation $V_{1/2}$,<br>**slower recovery from inactivation**
Q417P	GOF: hyperpolarized activation $V_{1/2}$,<br>slower inactivation time course,<br>**larger ramp current**,<br>larger window current<br>LOF: hyperpolarized inactivation $V_{1/2}$,<br>slower recovery from inactivation	GOF: hyperpolarized activation $V_{1/2}$,<br>slower inactivation time course,<br>larger window current<br>LOF: **smaller peak current density**,<br>hyperpolarized inactivation $V_{1/2}$,<br>slower recovery from inactivation
E713D	normal function	GOF: **hyperpolarized activation $V_{1/2}$**,<br>**faster recovery from inactivation**
R850Q	GOF: **hyperpolarized activation $V_{1/2}$**,<br>**depolarized inactivation $V_{1/2}$**,<br>larger persistent current,<br>larger window current<br>LOF: slower recovery from inactivation	GOF: **larger ramp current**,<br>larger persistent current,<br>larger window current.<br>LOF: **small peak current density**,<br>slower recovery from inactivation
G1475R	GOF: larger peak current density,<br>depolarized inactivation $V_{1/2}$,<br>slower inactivation time course,<br>**larger ramp current**,<br>larger persistent current	GOF: larger peak current density,<br>depolarized inactivation $V_{1/2}$,<br>slower inactivation time course,<br>larger persistent current
R1617L	GOF: depolarized inactivation $V_{1/2}$,<br>slower inactivation time course,<br>**larger ramp current**,<br>**larger persistent current**,<br>larger window current<br>LOF: smaller peak current density,<br>depolarized activation $V_{1/2}$	GOF: depolarized inactivation $V_{1/2}$,<br>slower inactivation time course,<br>larger window current<br>LOF: smaller peak current density,<br>depolarized activation $V_{1/2}$

Table S2 – *continued*

Variant	Functional effects in $Na_V1.6A$	Functional effects in $Na_V1.6N$
G1625R	GOF: depolarized inactivation $V_{1/2}$,<br>slower inactivation time course,<br>larger window current<br>LOF: smaller peak current density,<br>depolarized activation $V_{1/2}$	GOF: depolarized inactivation $V_{1/2}$,<br>slower inactivation time course,<br>larger window current<br>LOF: smaller peak current density,<br>depolarized activation $V_{1/2}$
I1631M	GOF: **depolarized inactivation $V_{1/2}$**,<br>**slower inactivation time course**,<br>**larger persistent current**,<br>larger window current<br>LOF: slower recovery from inactivation	GOF: larger window current<br>LOF: **smaller peak current density**,<br>slower recovery from inactivation
N1768D	GOF: depolarized inactivation $V_{1/2}$,<br>slower inactivation time course,<br>larger ramp current,<br>larger persistent current,<br>larger window current<br>LOF: depolarized activation $V_{1/2}$,<br>slower recovery from inactivation	GOF: depolarized inactivation $V_{1/2}$,<br>slower inactivation time course,<br>larger ramp current,<br>larger persistent current,<br>larger window current<br>LOF: depolarized activation $V_{1/2}$,<br>slower recovery from inactivation
N1877S	GOF: depolarized inactivation $V_{1/2}$,<br>slower inactivation time course,<br>**larger ramp current**	GOF: depolarized inactivation $V_{1/2}$,<br>slower inactivation time course
G1914S	GOF: slower inactivation time course	GOF: slower inactivation time course
T144S/S217P	Not relevant for $Na_V1.6A$	GOF: hyperpolarized activation $V_{1/2}$,<br>slower inactivation time course,<br>larger ramp current,<br>larger persistent current,<br>larger window current<br>LOF: hyperpolarized inactivation $V_{1/2}$,<br>smaller peak current density,<br>slower recovery from inactivation
T144S	GOF: slower inactivation time course,<br>larger ramp current,<br>larger window current	Not relevant for $Na_V1.6N$

Table S3. Comparison of reported functional properties of *SCN8A* variants.

Variant	Supplemental Reference	$Na_V1.6$ species	TTX-R	Isoform	β subunits	Cells	Peak Current density	Inactivation rate	Persistent current	Act $V_{1/2}$	Inact $V_{1/2}$	Ramp current	Recovery	Window current
R223G	Liu, et al., 2021 [21]	human	Y371C	5N	β1/β2	ND7/23	64%	NS	NS	-5.5 mV	-3.3 mV	NS	faster	larger
	de Koval, et al., 2014 [3]	mouse	Y371S	unknown	none	ND7/23	21-33%	NR	NR	NS	NS	larger	NR	NR
	This study	human	no	5A	none	ND7/LoNav	70%	NS	smaller	NS	-5.5 mV	smaller	slower	larger
R850Q	Pan, et al., 2020 [22]	human	Y371S	5N	none	ND7/23	NS	NS	larger	-4.6 mV	NS	NR	NS	larger
	This study	human	no	5N	none	ND7/LoNav	70%	NS	larger	NS	NS	larger	slower	larger
	This study	human	no	5A	none	ND7/LoNav	NS	NS	larger	-3.3 mV	+3.5 mV	NS	slower	larger
G1475R	Zaman, et al., 2019 [23]	human	no	5N	β1/β2	HEK	NS	NS	larger	NS	NS	NS	NR	NR
	Bayraktar, et al., 2021 [24]	human	Y371C	probably 5N	β1/β2	ND7/23	78%	slower	NS	NS	+5.2 mV	NS	NS	NR
	Liu, et al., 2019 [13]	human	Y371C	5N	β1/β2	ND7/23	NS	slower	NR	NS	+10.3 mV	NS	faster	NR
	This study	human	no	5N	none	ND7/LoNav	163%	slower	larger	NS	+8.2 mV	NS	NS	NS
	This study	human	no	5A	none	ND7/LoNav	142%	slower	larger	NS	+7.3 mV	larger	NS	NS
R1617L	Poulin, et al., 2021 [25]	human	no	unknown	β1	CHO	NS	slower	larger	NS	NS	NR	faster	NS
	Wagnon, et al., 2016 [26]	mouse	Y371S	5N	none	ND7/23	NS	slower	larger	-3.8 mV	+8.9 mV	NR	NR	larger
	This study	human	no	5N	none	ND7/LoNav	15%	slower	ND	+9.5 mV	+6.3 mV	ND	NS	larger
	This study	human	no	5A	none	ND7/LoNav	59%	slower	larger	+9.7 mV	+5.7 mV	larger	NS	larger
G1625R	Quinn, et al., 2024 [17]	human	Y371C	5N	β1/β2	Neuro-2a	~40	slower	larger	+12.3 mV	+26.9 mV	larger	NS	larger
	This study	human	no	5N	none	ND7/LoNav	46%	slower	ND	+5.3 mV	+3.8 mV	ND	NS	larger
	This study	human	no	5A	none	ND7/LoNav	28%	slower	ND	+10.7 mV	+10.4 mV	ND	NS	larger
N1768D	Veeramah, et al., 2012 [18]	mouse	Y371S	5N	none	ND7/23	44%	NR	larger	+4 mV	-11.6 mV	larger	NR	larger
	Patel, et al., 2016 [27]	human	no	5N	none	HEK	NS	slower	larger	NS	+12.6 mV	NR	slower	larger
	This study	human	no	5N	none	ND7/LoNav	NS	slower	larger	+4.8 mV	+11.4 mV	larger	slower	larger
	This study	human	no	5A	none	ND7/LoNav	NS	slower	larger	+5.0 mV	+13.1 mV	larger	slower	larger

NR = not reported; ND = not determined; NS = not significantly different from WT.

Table S4 – Mutagenic primer sequences for *SCN8A* variants (mutations are in bold red letters)

Variant	Primer	Primer sequence
V211A	Forward	TGACAGAGTTTG**C**GGACCTGGGCAATGTCTCAGCG
	Reverse	GTCC**G**CAAACTCTGTCACATATGCCATCATGATGAC
R223G	Forward	AACATTC**G**GGGTTCTCCGAGCTTTGAAAACTATCTCT
	Reverse	GGAGAACCC**C**GAATGTTCTCAGCGCTGAGACATTGCC
I231T	Forward	GAAAACTA**C**CTCTGTAATTCCAGGCCTGAAGACAATTG
	Reverse	TTACAGAG**G**TAGTTTTCAAAGCTCGGAGAACCCTGAATG
Y371S	Forward	CAGGACT**C**TTGGGAAAACTTGTATCAATTGA
	Reverse	AAGTTTTCCCAA**G**AGTCCTGGGTCATAAGGC
Y371C	Forward	CAGGACT**G**TTGGGAAAACTTGTATCAATTGA
	Reverse	AAGTTTTCCCAA**C**AGTCCTGGGTCATAAGGC
Q417P	Forward	ATGAAGAAC**C**GAATCAGGCAACACTGGAGGAGGC
	Reverse	CTGATTC**G**GTTCTTCATAAGCCATGGCCACCACAG
E713E	Forward	AGTAGAAGAACTGGA**C**GAGTCTCAGAGAAAGTGCCCGC
	Reverse	ACTC**G**TCCAGTTCTTCTACTAGTGTATTTGTAACAACA
G1914S	Forward	G**A**GCTTCATCTGCAAAAAGACAACTTCTAATAAGCTG
	Reverse	TTTTGCAGATGAAGC**T**CCGCCTTGCCAAATGTCCCCG
E713D	Forward	GTAGAAGAACTGGA**C**GAGTCTCAGAGAAAGTGCCCGCC
	Reverse	TC**G**TCCAGTTCTTCTACTAGTGTATTTGTAACAACACTC
R850Q	Forward	TGCTCC**A**AGTCTTCAAATTGGCCAAATCCTGGCCC
	Reverse	TTTGAAGACT**T**GGAGCAATCGGAAAGATCGCAGCAC
G1475R	Forward	CAAAAGAAAAAGTTC**A**GAGGTCAGGACATCTTCATGACCGAAG
	Reverse	C**T**GAACTTTTTCTTTTGTTGATTGAAGTTATCAATGATGACAC
R1617L	Forward	AACCCTATTCC**T**AGTCATCCGATTGGCCCGTATTGGG
	Reverse	TGACT**A**GGAATAGGGTTGGGGAAACAAAGTATTTCTC
G1625R	Forward	CCCGTATT**C**GGCGCATCTTGCGTCTGATCAAAGG
	Reverse	GATGCGCC**G**AATACGGGCCAATCGGATGACTCGG
I1631M	Forward	TGCGTCTGAT**G**AAAGGCGCCAAAGGGATTCGTACCC
	Reverse	GGCGCCTTT**C**ATCAGACGCAAGATGCGCCCAATACG
N1768D	Forward	TCCTGGAG**G**ACTTCAGTGTAGCCACAGAGGAAAGTGC
	Reverse	ACTGAAGT**C**CTCCAGGATGATGGCAATGTACATGTTCA
N1877S	Forward	ATCCA**G**TCCTTCCAAAGTGTCTTACGAGCCAATCA
	Reverse	CTTTGGAAGGA**C**TGGATGCCACGAACCGCTCTTCC
T144S	Forward	CTATTTTGA**G**CAACTGTGTATTCATGACTTTTAGT
	Reverse	TACACAGTTGCTCAAAATAGTGCACATAATGATCAT
S217P/R220R	Forward	TGTTC**CA**GCTCTA**CG**GACTTTCAGGGTACTGAGGGC
	Reverse	AAGTC**CG**TAGAGC**TG**GAACATTGCCTAGGTTTACAAAC

SUPPLEMENTAL REFERENCES

1. Epilepsy Genetics Initiative. De novo variants in the alternative exon 5 of SCN8A cause epileptic encephalopathy. *Genet Med.* 2018;20(2):275-81.
2. Truty R, Patil N, Sankar R, Sullivan J, Millichap J, Carvill G, et al. Possible precision medicine implications from genetic testing using combined detection of sequence and intragenic copy number variants in a large cohort with childhood epilepsy. *Epilepsia Open.* 2019;4(3):397-408.
3. de Kovel CG, Meisler MH, Brilstra EH, van Berkestijn FM, van 't SR, van LS, et al. Characterization of a de novo SCN8A mutation in a patient with epileptic encephalopathy. *Epilepsy Res.* 2014;108(9):1511-8.
4. Denis J, Villeneuve N, Cacciagli P, Mignon-Ravix C, Lacoste C, Lefranc J, et al. Clinical study of 19 patients with SCN8A-related epilepsy: Two modes of onset regarding EEG and seizures. *Epilepsia.* 2019;60(5):845-56.
5. Kong W, Zhang Y, Gao Y, Liu X, Gao K, Xie H, et al. SCN8A mutations in Chinese children with early onset epilepsy and intellectual disability. *Epilepsia.* 2015;56(3):431-8.
6. Wang J, Gao H, Bao X, Zhang Q, Li J, Wei L, et al. SCN8A mutations in Chinese patients with early onset epileptic encephalopathy and benign infantile seizures. *BMC Med Genet.* 2017;18(1):104.
7. Lindy AS, Stosser MB, Butler E, Downtain-Pickersgill C, Shanmugham A, Retterer K, et al. Diagnostic outcomes for genetic testing of 70 genes in 8565 patients with epilepsy and neurodevelopmental disorders. *Epilepsia.* 2018;59(5):1062-71.
8. Schreiber JM, Tochen L, Brown M, Evans S, Ball LJ, Bumbut A, et al. A multi-disciplinary clinic for SCN8A-related epilepsy. *Epilepsy Res.* 2020;159:106261.
9. Parrini E, Marini C, Mei D, Galuppi A, Cellini E, Pucatti D, et al. Diagnostic Targeted Resequencing in 349 Patients with Drug-Resistant Pediatric Epilepsies Identifies Causative Mutations in 30 Different Genes. *Human Mutation.* 2017;38(2):216-25.
10. Gardella E, Marini C, Trivisano M, Fitzgerald MP, Alber M, Howell KB, et al. The phenotype of SCN8A developmental and epileptic encephalopathy. *Neurology.* 2018;91(12):e1112-e24.
11. Xiao Y, Xiong J, Mao D, Liu L, Li J, Li X, et al. Early-onset epileptic encephalopathy with de novo SCN8A mutation. *Epilepsy Res.* 2018;139:9-13.
12. Johannesen KM, Gardella E, Encinas AC, Lehesjoki AE, Linnankivi T, Petersen MB, et al. The spectrum of intermediate SCN8A-related epilepsy. *Epilepsia.* 2019;60(5):830-44.

13. Liu Y, Schubert J, Sonnenberg L, Helbig KL, Hoei-Hansen CE, Koko M, et al. Neuronal mechanisms of mutations in SCN8A causing epilepsy or intellectual disability. *Brain.* 2019;142(2):376-90.
14. Ohba C, Kato M, Takahashi S, Lerman-Sagie T, Lev D, Terashima H, et al. Early onset epileptic encephalopathy caused by de novo SCN8A mutations. *Epilepsia.* 2014;55(7):994-1000.
15. Larsen J, Carvill GL, Gardella E, Kluger G, Schmiedel G, Barisic N, et al. The phenotypic spectrum of SCN8A encephalopathy. *Neurology.* 2015;84(5):480-9.
16. Fitzgerald TW, Gerety SS, Jones WD, van Kogelenberg M, King DA, McRae J, et al. Large-scale discovery of novel genetic causes of developmental disorders. *Nature.* 2015;519(7542):223-8.
17. Quinn S, Zhang N, Fenton TA, Brusel M, Muruganandam P, Peleg Y, et al. Complex biophysical changes and reduced neuronal firing in an SCN8A variant associated with developmental delay and epilepsy. *Biochim Biophys Acta Mol Basis Dis.* 2024;1870(5):167127.
18. Veeramah KR, O'Brien JE, Meisler MH, Cheng X, Dib-Hajj SD, Waxman SG, et al. De novo pathogenic SCN8A mutation identified by whole-genome sequencing of a family quartet affected by infantile epileptic encephalopathy and SUDEP. *Am J Hum Genet.* 2012;90:502-10.
19. Anand G, Collett-White F, Orsini A, Thomas S, Jayapal S, Trump N, et al. Autosomal dominant SCN8A mutation with an unusually mild phenotype. *Eur J Paediatr Neurol.* 2016;20(5):761-5.
20. Butler KM, da Silva C, Shafir Y, Weisfeld-Adams JD, Alexander JJ, Hegde M, et al. De novo and inherited SCN8A epilepsy mutations detected by gene panel analysis. *Epilepsy Res.* 2017;129:17-25.
21. Liu Y, Koko M, and Lerche H. A SCN8A variant associated with severe early onset epilepsy and developmental delay: Loss- or gain-of-function? *Epilepsy Res.* 2021;178:106824.
22. Pan Y, and Cummins TR. Distinct functional alterations in SCN8A epilepsy mutant channels. *J Physiol.* 2020;598(2):381-401.
23. Zaman T, Abou Tayoun A, and Goldberg EM. A single-center SCN8A-related epilepsy cohort: clinical, genetic, and physiologic characterization. *Ann Clin Transl Neurol.* 2019;6(8):1445-55.
24. Bayraktar E, Liu Y, Sonnenberg L, Hedrich UBS, Sara Y, Eltokhi A, et al. In vitro effects of eslicarbazepine (S-licarbazepine) as a potential precision therapy on SCN8A variants causing neuropsychiatric disorders. *Br J Pharmacol.* 2023;180(8):1038-55.
25. Poulin H, and Chahine M. R1617Q epilepsy mutation slows $Na_V1.6$ sodium channel inactivation and increases the persistent current and neuronal firing. *J Physiol.* 2021;599(5):1651-64.

26. Wagnon JL, Barker BS, Hounshell JA, Haaxma CA, Shealy A, Moss T, et al. Pathogenic mechanism of recurrent mutations of SCN8A in epileptic encephalopathy. *AnnClinTranslNeurol.* 2016;3(2):114-23.

27. Patel RR, Barbosa C, Brustovetsky T, Brustovetsky N, and Cummins TR. Aberrant epilepsy-associated mutant $Na_V1.6$ sodium channel activity can be targeted with cannabidiol. *Brain.* 2016;139(Pt 8):2164-81.
