# Supplementary material for: Molecular and cellular context influences SCN8A variant function
Source: JCI Insight. 2024 May 21;9(12):e177530. doi: 10.1172/jci.insight.177530 (PMC11383174; doi:10.1172/jci.insight.177530)
Supplement: Unedited blot and gel images [file jciinsight-9-177530-s104.pdf]

Fig. 1A full unedited blots*

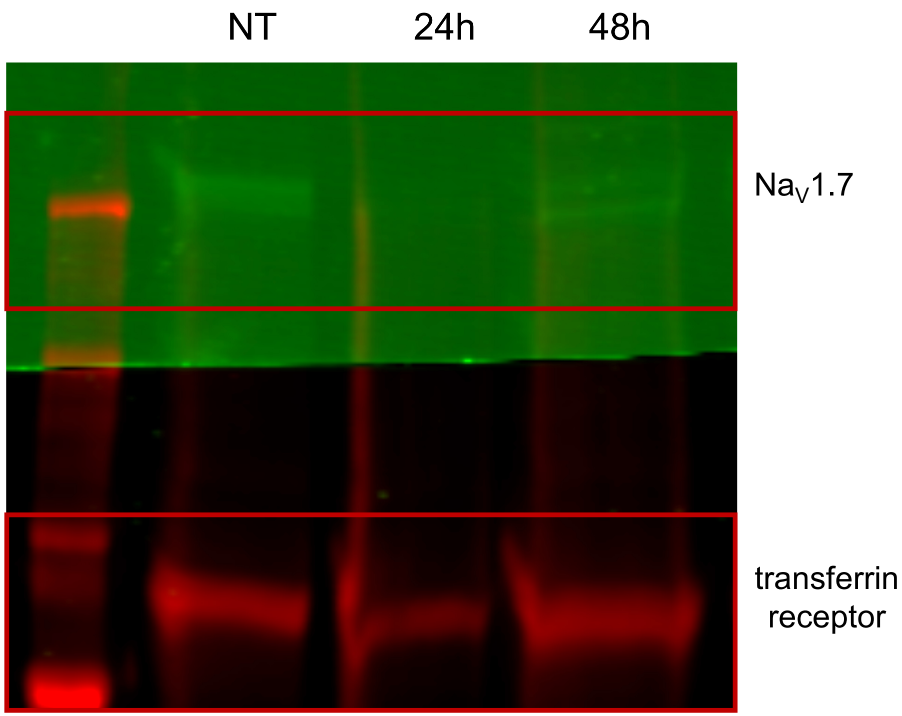

- The blot was cut prior to applying primary antibodies.
- The image was converted to greyscale for the article.

Fig. 1C full unedited blots

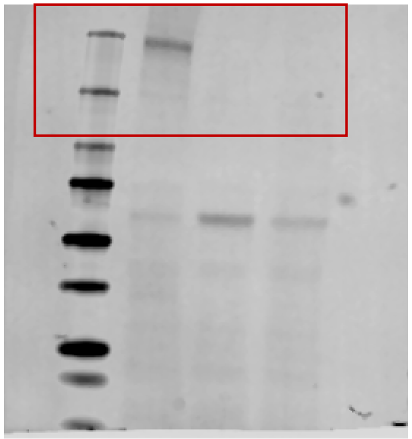

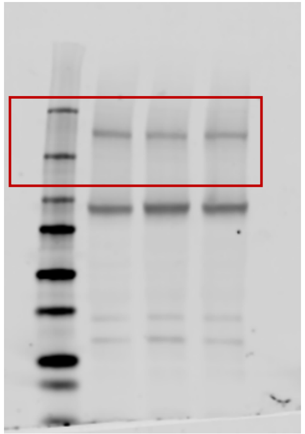

Lane 1 – ND723
Lane 2 – ND7/LoNav clone 2-11
Lane 3 – ND7/LoNav clone 6-2
